# NAP1L1 Functions as a Novel Prognostic Biomarker Associated With Macrophages and Promotes Tumor Progression by Influencing the Wnt/β-Catenin Pathway in Hepatocellular Carcinoma

**DOI:** 10.3389/fgene.2022.876253

**Published:** 2022-05-19

**Authors:** Bingbing Shen, Wenjie Zhu, Xinyuan Liu, Jianxin Jiang

**Affiliations:** ^1^ Department of Hepatobiliary Surgery, Renmin Hospital of Wuhan University, Wuhan, China; ^2^ Department of Hepatic–Biliary-Pancreatic Surgery, The Affiliated Hospital of Guizhou Medical University, Guiyang, China

**Keywords:** NAP1L1, Bioinformatics, cell cycle, Wnt/β-catenin, immune, hepatocellular carcinoma, clinical

## Abstract

Hepatocellular carcinoma (HCC) is regarded as one of the universal cancers in the world. Therefore, our study is based on clinical, molecular mechanism and immunological perspectives to analyze how *NAP1L1* affects the progression of HCC. To begin with, the gene expression datasets and clinical data of GSE14520, GSE76427, ICGC, and TCGA are originated from GEO, ICGC, and TCGA databases. Subsequently, DEG screening was performed on data using R studio, and we finally found that 2,145 overlapping DEGs were screened from four datasets at the end. Then, we used R studio to filter the survival-related genes of the GSE76427 and ICGC datasets, and we screened out 101 survival-related genes. Finally, 33 common genes were screened out from 2,145 overlapping DEGs and 101 survival-related genes. Then, *NAP1L1* was screened from 33 common genes using the CytoHubba plug-in in Cytoscape software. Furthermore, ground on GEO, ICGC, and TCGA databases, the survival analysis, clinical feature analysis, univariate/multivariate regression analysis, and multiple GSEA were used to study NAP1L1. The Conclusion claimed that HCC patients with higher expression levels of NAP1L1 had a poorer prognosis than those with lower expression levels. Thus, we believe that *NAP1L1* is an independent prognostic factor for HCC. In order to shed light on NAP1L1’s molecular mechanism promoting the progression of HCC closely, the GSEA tool was applied to complete the GSEA of the four datasets. Furthermore, the results confirmed that *NAP1L1* could promote HCC progression by regulating the G2/M transition of the cell cycle and *Wnt* signaling pathway. Western blot and flow cytometry were also performed to understand those mechanisms in this study. The result of Western blot showed that *NAP1L1* silencing led to downregulation of *CDK1* and *β-catenin* proteins; the result of flow cytometry showed that cell numbers in the G2 phase were significantly increased when *NAP1L1* was silenced. Thus, we claimed that *NAP1L1* might promote HCC progression by activating the *Wnt* signaling pathway and promoting cell cycle G2/M transition. In addition, ground on GSE14520 and GSE76427 datasets, and ICGC and TCGA databases, the correlation between *NAP1L1* and immune cells was analyzed in HCC patients. At the same time, the TISIDB online database and the TIMER online database were testified to the association between *NAP1L1* and immune cells. Hence, the summary shows that *NAP1L1* was connected with a certain amount of immune cells. We can speculate that *NAP1L1* may influence macrophages to promote HCC progression through some potential mechanisms.

## 1 Introduction

According to the 2020 world cancer data, the incidence of HCC ranked the seventh, and the mortality rate related to HCC ranked the fourth in the world (Sung et al., 2021). Surgical and non-surgical therapies are the primary therapy methods used for treating HCC. The surgical treatment mainly covers liver resection, liver transplantation, and radiofrequency ablation, and the non-surgical treatment is based on targeted immunotherapy such as sorafenib and monoclonal antibodies (Chen et al., 2020). Although there are tremendous treatment methods for HCC, the extreme unbalance of survival and recurrence of HCC patients is a significant difficulty to treat HCC now. Therefore, we place more value on studying the potential molecular mechanisms of HCC progression for working on the perfection of the strategies of a comprehensive therapy.

**FIGURE 1:**
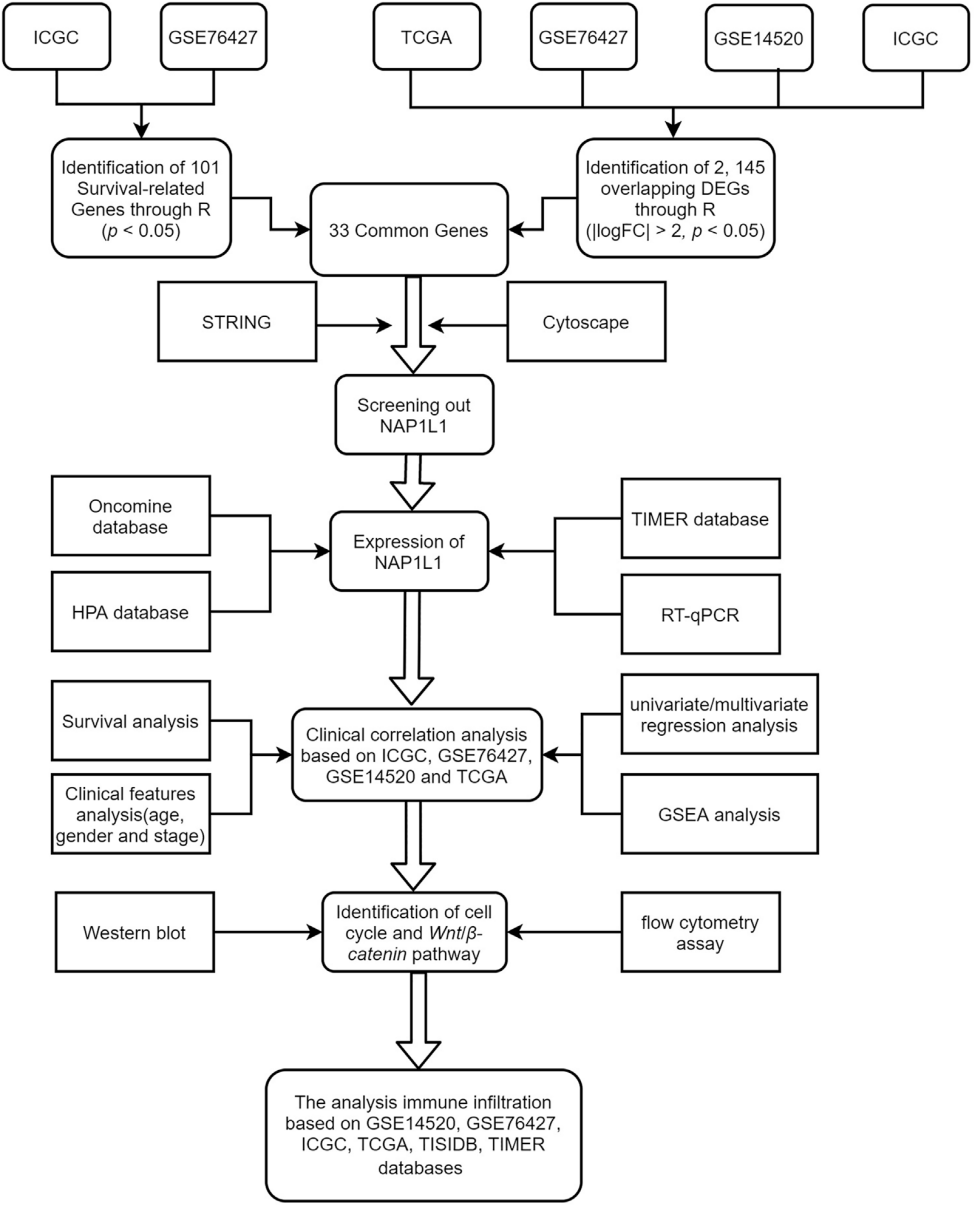
Flow diagram for research.

Bioinformatics has become a hot medical analysis tool to filtrate key genes. GSE14520, GSE76427, ICGC, and TCGA gene expression datasets, and clinical data were obtained from GEO, ICGC, and TCGA databases. Then, we searched for DEGs ground on the GEO microarray, ICGC, and TCGA datasets and screened survival-related genes in the GSE76427 and the ICGC datasets through R studio. Next, we obtained 33 common genes between the 2,145 DEGs and the 101 survival-related genes through the Venn online tool. Subsequently, we analyzed the 33 common genes through a PPI network. Cytoscape software, including the MCODE and the CtyoHubba plug-ins, screened out the essential modules by the PPI network and hub genes among all common genes. GEO, ICGC, and TCGA databases illustrated the correlation of hub genes with the survival and prognosis of HCC patients. The intrinsic mechanisms that hub genes use to influence HCC progression were found by analyzing multiple databases. Finally, some experiments based on its potential mechanism were carried out. Although most of our studies were based on bioinformatics analysis, this research is critical and crucial for accelerating medical progression and making a minor contribution to dig the occurrence mechanisms of HCC in the medical area. The workflow of the specific analysis is shown in Figure 1.

## 2 Methods and Materials

### 2.1 The Acquisitions of Gene Expression and Clinical Data

The gene expression and clinical data covering age, gender, stage, and survival data were rooted in the GEO (https://www.ncbi.nlm.nih.gov/geo/), ICGC (https://icgc.org/), and TCGA databases (https://tcga-data.nci.nih.gov/). Gene expression and clinical data of the GSE14520 series (Roessler et al., 2010) and the GSE76427 series (Grinchuk et al., 2018) originated from the GEO database. Gene expression and clinical data of GSE14520 based on the GPL3921 platform [HT_HG-U133A] (Affymetrix HT Human Genome U133A Array), including 241 non-tumor specimens and 247 tumor specimens (including 227 paired HCC specimens). Gene expression and clinical data of GSE76427 based on the GPL10558 platform (Illumina HumanHT-12 V4.0 expression bead chip), including 52 non-tumor specimens and 115 tumor specimens (including 52 paired HCC specimens). The gene expression files (ICGC-LIRI-JP) of the ICGC database based on the Illumina HiSeq RNA Seq platform, including 202 non-tumor specimens and 243 tumor specimens (including 202 paired HCC specimens), were obtained from the ICGC database. The RNA sequencing and clinical data (TCGA-LIHC-FPKM) based on the Illumina HiSeq RNA-Seq platform, including 50 non-tumor specimens and 374 tumor specimens (including 50 paired HCC specimens), were obtained from the TCGA database.

### 2.2 The Identification of DEGs and Survival-Related Genes, and Screening out Common Genes

The DEGs of non-tumor specimens and tumor specimens were identified with R studio by dealing with the raw data of GSE14520, GSE76427, ICGC, and TCGA databases. In the four datasets, the RMA package was utilized to standardize the entire raw data, the Affy package was utilized to evaluate the quality of data, and the Limma package was utilized to filtrate DEGs whose standard was deemed as the | logFC| > 2 as well as the adjusted *p* < 0.05. In addition, the survival package of R studio was also utilized to filtrate survival-related genes from GSE76427 and ICGC, in which the cutoff criterion was *p*-values < 0.05. The common genes between DEGs and survival-related genes were selected by R studio, painting a Venn diagram.

### 2.3 PPI Network and Screening out Hub Gene

The STRING database (http://string-db.org)(Szklarczyk et al., 2015) formed the PPI network where the MCODE plug-in chose the essential modules in the Cytoscape application. In addition, the standards of choosing the essential modules are k-core = 2, degree cutoff = 2, node score cutoff = 0.2, and maximum depth = 100. CytoHubba plug-in was another plug-in in Cytoscape software in which the top 10 hub genes were selected in the PPI network. What is more, the 12 calculating methods in CytoHubba presented as the following: DMNC, ClusteringCoefficient, EPC, Degree, BottleNeck, EcCentricity, MNC, Radiality, Betweenness, MCC, Stress, and Closeness, which generated 12 different outcomes; through comparing with them, the *NAP1L1* was chosen as the objective of our study.

### 2.4 The Analysis of the Oncomine Database and HPA Database

The Oncomine database (https://www.oncomine.org/resource/login.html)(Rhodes et al., 2007) consists of 86,733 specimens and 715 gene expression data. Moreover, it is a unitary integrated database aimed to accelerate data mining efforts effectively. Therefore, this database evaluated the *NAP1L1* expression in human tumors. Accordingly, HPA (https://www.proteinatlas.org) is deemed a kind of online tool whose goal was to elucidate the level of *NAP1L1* protein exiting in non-tumor tissues and HCC tissues (Pontén et al., 2011). In 2003, the Swedish-based program, the HPA, witnessed the world project of human proteins. Specifically, the cells, tissues, and organs applying the combination of diverse omics technologies containing antibody-based imaging were all taken in human proteins. HPA version 21.0, appearing in November 2021, was used in this study (Uhlén et al., 2015; Thul et al., 2017; Uhlen et al., 2017). The immunohistochemistry pictures originated from HPA. The expression of the *NAP1L1* protein was explained in non-tumor tissues and HCC tissues among HCC patients.

### 2.5 Survival Analysis, Clinical Feature Analysis, Univariate/Multivariate Regression Analysis, and GSEA

R studio was utilized to achieve survival analysis, clinical feature analysis, and univariate/multivariate regression analysis. Patients’ gene expression datasets and clinical data were used in this study, including GSE14520, GSE76427, ICGC, and TCGA datasets. Nine packages are being taken in this research: the beeswarm, Limma, survival, survminer, Ggpubr, plyr, grid, ggplot2, and gridExtra packages. To be more specific, the beeswarm and Limma packages were used to conduct the differential expression analysis, the survival package and survminer package were applied to perform the survival analysis, the survival package also was utilized to do the univariate/multivariate regression analysis, the Ggpubr package was utilized to carry out the analysis of the clinical features, and the plyr, ggplot2, grid, and gridExtra packages were performed to analyze the multiple GSEA.

GSEA4.2.1 application was derived from the Broad Institute (http://www.gsea-msigdb.org/gsea/login.jsp) (Subramanian et al., 2005), which still played a significant role in generating enrichment results. Plus, R studio was visualized well to complete multiple GSEA results.

### 2.6 Immune Cell Infiltration Analysis and IPS

CIBERSORT was utilized to figure out the enrichment files of immune cells in the HCC sample ground on GSE14520, GSE76427, ICGC, and TCGA (Chen B. et al., 2018). TISIDB (http://cis.hku.hk/TISIDB/index.php) was utilized to detect the interactions between 28 types of TILs and 30 types of cancers in humans to check the tumor–immune system interactions (Ru et al., 2019). The association between *NAP1L1* and TILs was tested through Spearman’s test, in which all hypothetical tests were deemed as statistically significant in that they were bilateral and *p*-value < 0.05. Then, to verify the enrichment of six immune cells (dendritic cells, B cells, CD4^+^T cells, CD8^+^T cells, macrophages, and neutrophils) in HCC patients, the TIMER online database was used to verify the relations between *NAP1L1* expression and the level of immune infiltration (Li T. et al., 2017).

IPS is a terminology that comprises MHC molecules, immunomodulators, effector cells, and suppressor cells. According to the expression of representative genes, IPS could be figured out when applying it, and the TCIA (https://tcia.at/home) was applied to acquire the IPS about HCC patients. R studio was utilized to achieve immune cell infiltration analysis. The immune cell infiltration analysis needed Limma, ggExtra, vioplot, ggpubr, ggplot2, and Venn Diagram packages. Specifically, the Limma package was used to acquire the results of CIBERSORT, and the vioplot package was utilized to paint the violin plot. Moreover, ggpubr, ggplot2, ggpubr, and ggExtra were applied to perform the correlation analysis between NAP1L1’s expression and the immune cell, and to paint the correlation graph; the Venn Diagram package was used to paint the Venn graphs.

### 2.7 Cell Culture

As the objective of our study, the normal liver cell lines (MIHA) and the HCC cell lines (MHCC-LM3, PRF-5, Huh-7, MHCC-97H, and Hep-G2) are originated from the cell bank of the Chinese Academy of Sciences (Shanghai, China). Furthermore, they were maintained in a DMEM cell culture (Gibco, New York, United States) supplemented with 10% FBS (Gemini, West Sacramento, United States) in the incubator at 37°C with 5% CO2.

### 2.8 Cell Transfection

NAP1L1 siRNA was synthesized from RiboBio (Guangzhou, China) and was used to reduce the NAP1L1 expression. MHCC-97H and Huh-7 cells were cultivated into each well of the six-well plates until they were 30–60% confluent. After that, cells were washed, placed in a serum-free medium, and transfected with siRNA using Lipofectamine™2000 according to the manufacturer’s instructions (Invitrogen, MA, United States). After 6 h, the medium was changed to a complete medium, and cells were cultured at 37°C in 5% CO_2_. Four groups were generated for all experiments, a negative control group (negative control); a siRNA1 group (siRNA1); a siRNA2 group (siRNA2); a siRNA3 group (siRNA3).

### 2.9 qRT-PCR

The whole RNA was obtained at first by utilizing the TRIzol reagent and then going through the reverse transcription way to reach the cDNA under the guidance of the Thermo Fisher Scientific Reagent Kit. Finally, we did a qRT-PCR reaction of cDNA, taking a Bio-Rad qRT-PCR Checking System.

### 2.10 Western Blot

First of all, we centrifuged and gathered the cells when cells were transfected after 48 h. Then, the radioimmunoprecipitation assay buffer was applied to acquire the whole-cell protein lysates. Furthermore, detaching by SDS-PAGE, the protein lysates were blotted into PVF membranes (Bio-Rad). The principal antibodies, including *NAP1L1* (14898-1-AP; 45kDa; 1:1000), *CDK1* (19532-1-AP; 34kDa; 1:1000), *β-catenin* (19532-1-AP; 45kDa; 1:1000), and *GAPDH* (10494-1-AP; 36kDa; 1:1000), were attached to the membranes after blocking, which they stored one night at 4°C. Next, after the TBST was washed three times, the second antibody was hatched for 2 h at homeothermy, and then TBST was eluted three times once again. In the end, the Western blot was visualized by imaging systems.

### 2.11 The Analysis of the Cell Cycle

The day before the transfection, MHCC-97H and Huh-7 cells were planted into the culture plates. When they were transfected successfully, those cells were eluted with PBS and fitted in 75% pre-cooling alcohol. Before applying the flow cytometer, MHCC-97H and Huh-7 cells were hatched in 500 μl specimen buffer and 0.25 mg/ml of RNase A, maintaining thirty minutes at homeothermy. Subsequently, BD FACSCalibur Flow Cytometer was utilized to count the quantities of the 2 cells in each stage, and FlowJo was applied to explain these data.

### 2.12 Statistical Analysis

In terms of the statistical analysis, we considered the two software: GraphPad Prism (version: 8.2.1) and R studio (version:4.0.5). Data analysis was regarded as meaningful data, while the formula *p* < 0.05 showed the meaning of a statistically significant difference.

## 3 Results

### 3.1 Screening out Key Gene *NAP1L1*

**FIGURE 2:**
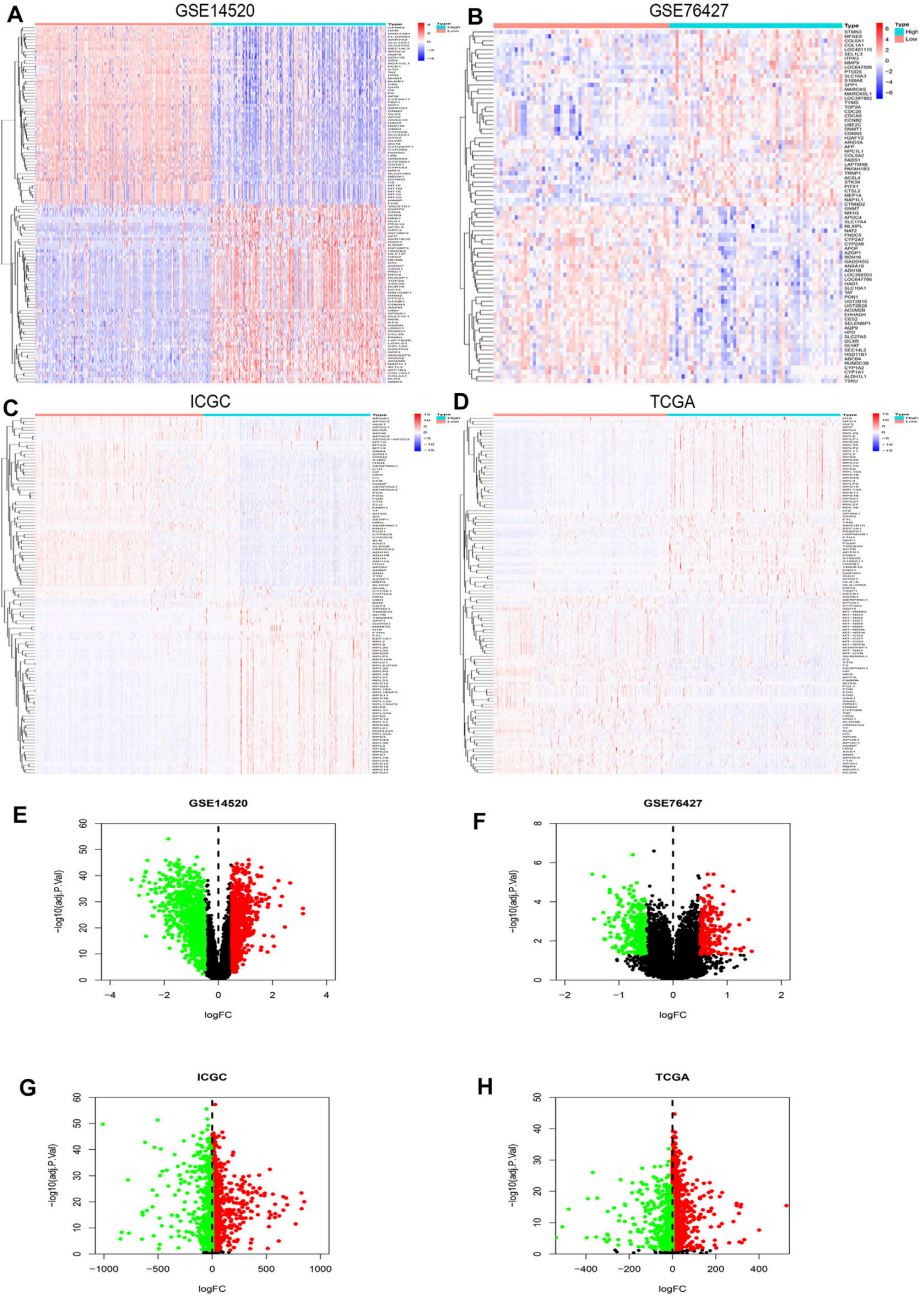
DEGs’ heatmap and volcano plot in GSE14520, GSE76427, ICGC, and TCGA: **(A)** The heatmap in GSE14520, **(B)** the heatmap in GSE76427, **(C)** the heatmap in ICGC, **(D)** the heatmap in TCGA, **(E)** the volcano plot in GSE14520, **(F)** the volcano plot in GSE76427, **(G)** the volcano plot in ICGC, and **(H)** the volcano plot in TCGA. In the heatmaps, the high expression represented red, and blue represented the low expression. Red showed the upregulation genes in the volcano plots, and green presented the downregulation genes. In addition, the black dots were not the differential gene in expression in the volcano plots.

**FIGURE 3:**
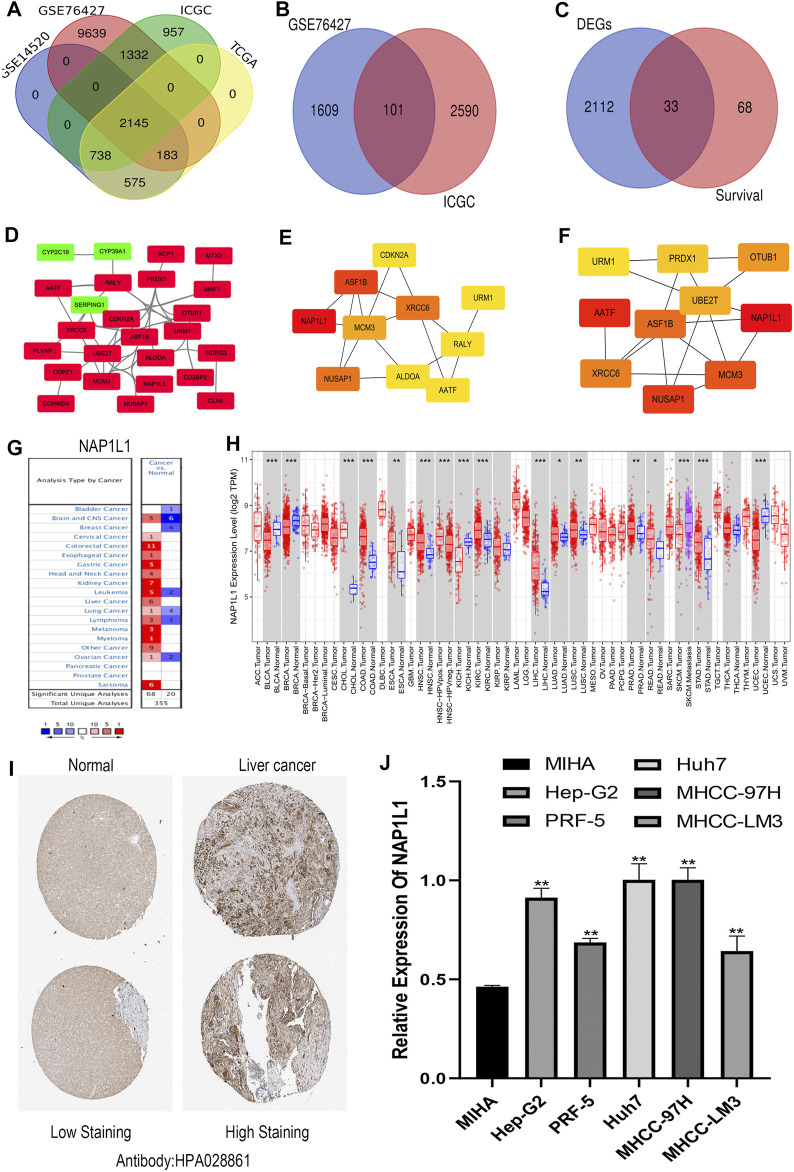
Screening out the hub gene *NAP1L1*, and *NAP1L1* was up-regulated in HCC. **(A)** The identification of the 2,145 overlapping DEGs ground on GSE14520, GSE76427, ICGC, and TCGA. **(B)** The identification of the 101 overlapping survival-related genes ground on GSE76427 and TCGA. **(C)** The identification of the 33 common genes. **(D)** The PPI network was constructed using the 33 common genes. **(E)** The PPI network of the top 10 hub genes *via* DMNC. **(F)** The PPI network of the top 10 hub genes *via* ClusteringCoefficient. **(G)** The expression of *NAP1L1* applying the Oncomine database. **(H)** The expression of *NAP1L1* applying the TIMER database (*p < 0.05; **p < 0.01; ***p < 0.001). **(I)** The expression of *NAP1L1* applying the HPA database. **(J)** The mRNA expression of *NAP1L1* applying the RT-qPCR.

R studio demonstrated the DEGs in every database (3,641 in GSE14520, 13,299 in GSE76427, 5,172 in ICGC, and 3,642 in TCGA) and DEGs whose standard was deemed as the | logFC| > 2 as well as the adjusted *p* < 0.05. At the same time, R studio was also used to paint heatmaps and volcano plots about DEGs in every database, presented in figures (Figures 2A–H). The 2,145 overlapping DEGs were examined in the aforementioned databases, and the results were displayed in a Venn graph (Figure 3A). In addition, we continued to do a survival filter using R studio (1,710 in GSE76427 and 2,691 in ICGC), and the cutoff criterion was *p*-values < 0.05. Therefore, 101 survival-related genes were screened out. The outcomes were displayed in a Venn graph (Figure 3B). Furthermore, we intersected 33 common genes from DEGs and survival-related genes whose outcomes were displayed in a Venn graph (Figure 3C).

The STRING was applied to produce a PPI network including 33 common genes taken by the Cytoscape application. It covered 26 high-expression genes labeled with red color and seven low-expression genes labeled with green color. Here, we only showed 25 genes related to each other (Figure 3D). CytoHubba plug-in functions as a plug-in in Cytoscape application were selected the top 10 hub genes in the PPI network. The 12 calculating methods in cytoHubba presented as the following: DMNC, ClusteringCoefficient, EPC, Degree, BottleNeck, EcCentricity, MNC, Radiality, Betweenness, MCC, Stress, and Closeness, which generated 12 various results in the top 10 key genes through comparing with them. Moreover, we claimed that *NAP1L1* obtained the highest score in DMNC (Figure 3E) and ClusteringCoefficient (Figure 3F) so that the *NAP1L1* was chosen as the objective of our study.

### 3.2 Expression of *NAP1L1* in HCC

Gene expression analyses using the ONCOMINE and the TIMER claimed that *NAP1L1* mRNA levels were evidently higher in HCC than in non-tumor tissues (Figures 3G,H). The HPA database presented the expression levels of the *NAP1L1* protein in HCC. The levels of the *NAP1L1* protein were low in normal liver tissues, and the high expression levels of *NAP1L1* were observed in HCC tissues (Figure 3I). Subsequently, the qRT-PCR measured the *NAP1L1* mRNA level (Figure 3J). Thus, the expression of *NAP1L1* was higher based on the normal liver tissue in HCC tissues.

### 3.3 Survival Analysis, Clinical Feature Analysis, Univariate/Multivariate Regression Analysis, and Multiple GSEA of *NAP1L1* in GSE14520 Datasets, GSE76427 Datasets, ICGC Datasets, and TCGA Datasets

**FIGURE 4:**
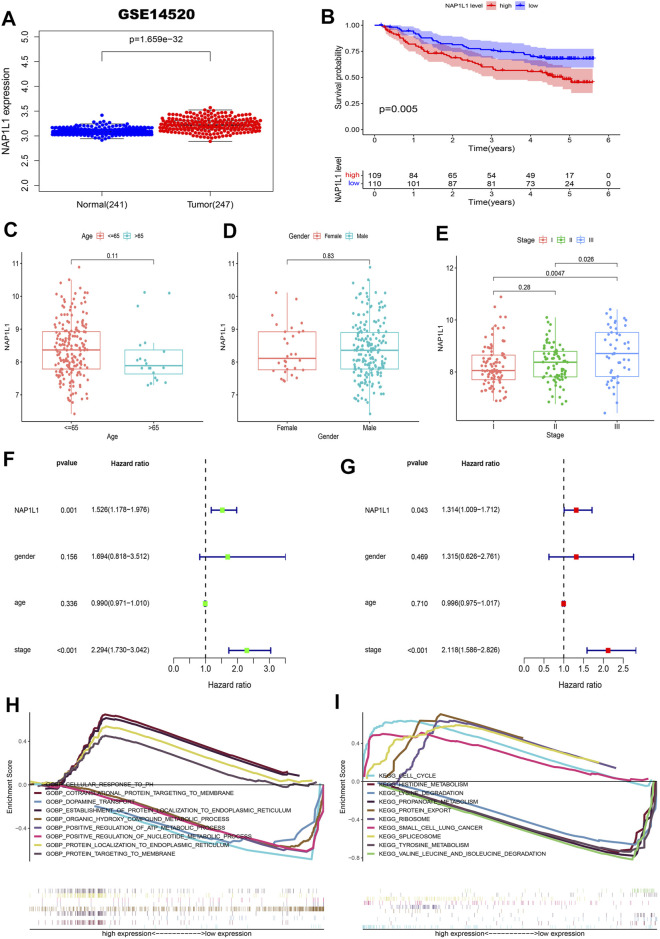
Survival analysis, the clinical feature analysis, the univariate/multivariate regression analysis, and the multiple GSEA in the GSE14520 datasets. **(A)** The scatterplot of the *NAP1L1* expression in the non-tumor and HCC specimens. **(B)** The survival curve of OS in the high and the low *NAP1L1* expression groups. **(C)** The bar graph in age. **(D)** The bar graph in gender. **(E)** The bar graph in stage. **(F)** The univariate regression analysis. **(G)** The multivariate regression analysis. **(H)** The multiple GSEA ground on the GO. **(I)** The multiple GSEA ground on the KEGG.

First, the gene expression profile and the clinical information (the GSE14520, GSE76427 datasets, ICGC datasets, and TCGA datasets) were obtained from the GEO, ICGC, and TCGA databases. From those scatterplots (Figure 4A, Supplementary Figures S1A,2A, 3A), we concluded that the *NAP1L1* expression of HCC specimens was evidently higher than that of the non-tumor specimens (*p* < 0.05). From those survival curves (Figure 4B, Supplementary Figures S1B,2B, 3B), we found that the high expression of *NAP1L1* is closely associated with a bad OS (*p* < 0.05).

Furthermore, we also sought the correlations between the expression of *NAP1L1* and the HCC patients’ clinical features from age (Figure 4C, Supplementary Figures S1C,S2C, S3C), gender (Figure 4D, Supplementary Figures S1D,S2D, S3D), and stage (Figure 4E, Supplementary Figures S1E,S2E, S3E). Those results showed a significant difference between the expression of *NAP1L1* and the stage of HCC patients (*p* < 0.05). The Conclusion also showed that the significant difference did not exist in age (*p* > 0.05) and gender (*p* > 0.05). Then, the univariate (Figure 4F, Supplementary Figures S1F,S2F, S3F) and the multivariate regression analyses (Figure 4G, Supplementary Figures S1G,S2G, S3G) were performed with the clinical data of HCC patients, which reveals that *NAP1L1* was statistically significant in the univariate regression analysis (*p* < 0.05) and the multivariate regression analysis (*p* < 0.05). This result claimed that *NAP1L1* was an independent prognostic factor in HCC patients.

Finally, to deeply investigate how the *NAP1L1* promotes HCC progression, the HCC patients were separated into the high and low expression groups ground on the expression of *NAP1L1*. Furthermore, we took multiple GSEA employing the GO and the KEGG, and the enrichment results of the top five genes of upregulation and the top five genes of downregulation were displayed in GO and KEGG gene sets (Figures 4H,I, Supplementary Figures S1H, S1I, S2H, S2I, S3H, S3I).

In Conclusion, the aforementioned results in GSE14520 datasets, GSE76427 datasets, ICGC datasets, and TCGA datasets showed that NAP1L1 is a high expression in HCC tissue and is closely associated with poorer prognosis of HCC patients. In addition, we found correlations between the expression of NAP1L1 and clinical features in HCC patients, and NAP1L1 is an independent prognostic factor in HCC patients. which could promote the progression of HCC by some complex mechanisms.

### 3.4 The Cell Cycle and Wnt Signaling Pathway Through GSEA in GSE14520 Datasets, GSE76427 Datasets, ICGC Datasets, and TCGA Datasets

**FIGURE 5:**
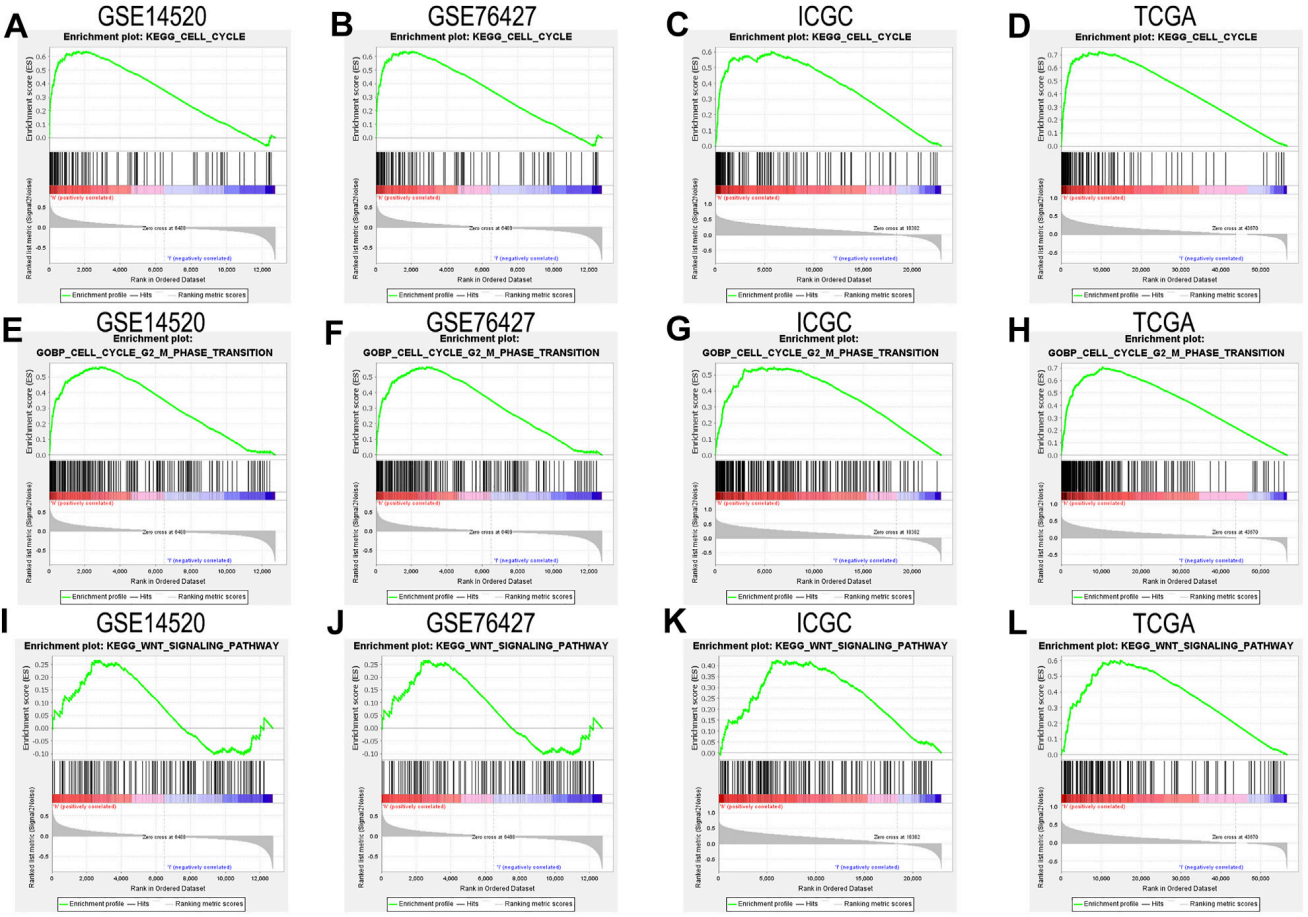
The GSEA: **(A)** the GSEA ground on KEGG in the GSE14520 dataset, **(B)** the GSEA ground on KEGG in the GSE76427 dataset, **(C)** the GSEA ground on KEGG in the ICGC dataset, **(D)** the GSEA ground on KEGG in the TCGA dataset, **(E)** the GSEA ground on GO in the GSE14520 dataset, **(F)** the GSEA ground on GO in the GSE76427 dataset, **(G)** the GSEA ground on GO in the ICGC dataset, **(H)** the GSEA ground on GO in the TCGA dataset, **(I)** the GSEA ground on KEGG in the GSE14520 dataset, **(J)** the GSEA ground on KEGG in the GSE76427 dataset, **(K)** the GSEA ground on KEGG in the ICGC dataset, and **(L)** the GSEA ground on KEGG in the TCGA dataset.

We closely understood the aforementioned GSEA results to explore complex mechanisms of *NAP1L1* that promoted HCC progression. Hence, we further found that cell cycle and *Wnt* signaling pathway can be found in the GSE14520 dataset, GSE76427 dataset, ICGC dataset, and TCGA dataset, based on GSEA. The result demonstrated that *NAP1L1* might accelerate the progression of HCC, which relies on the cell cycle (Figures 5A–D), especially the G2/M phase transition (Figures 5E–H) and Wnt signaling pathway (Figure 5I–L).

### 3.5 ***NAP1L1*** Relevant to the G2/M Phase Transition of the Cell Cycle and Wnt Signaling Pathway in MHCC-97H and Huh7 Cell Lines

**FIGURE 6:**
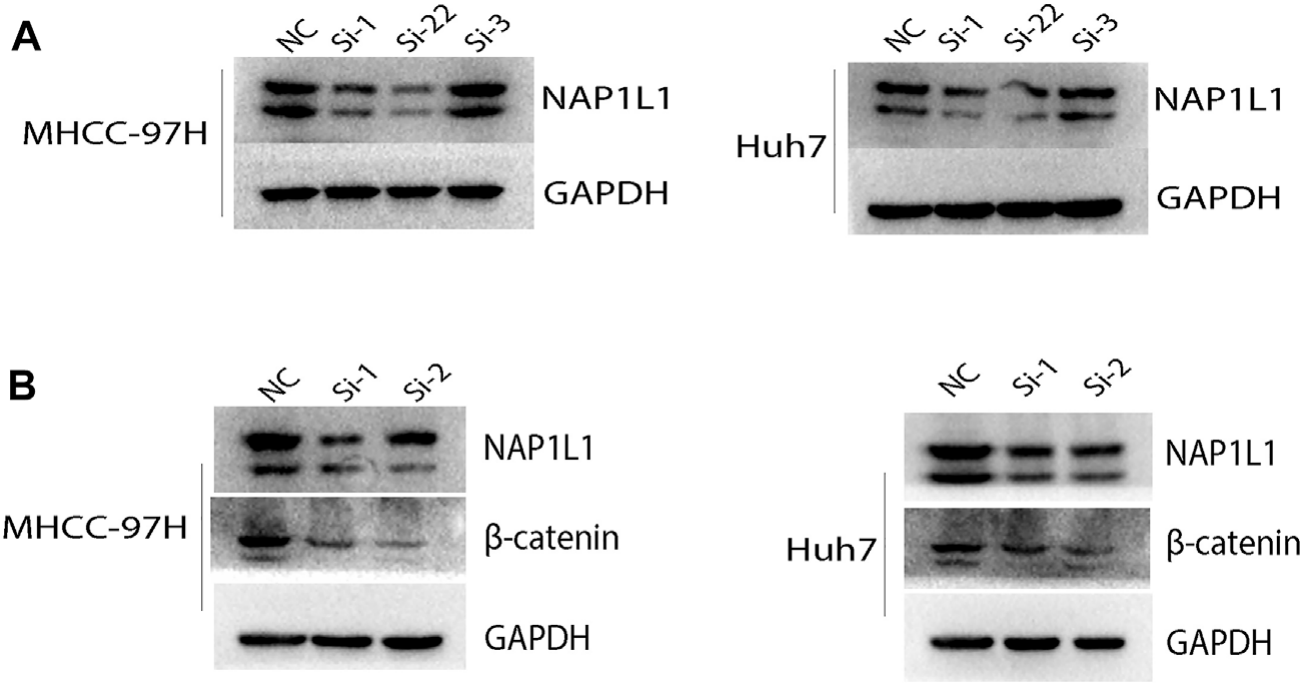
The Western blot: **(A)** the Western blot was utilized to check the expression level of *NAP1L1* among the NC, si-1, si-2, and si-3 groups in MHCC-97H and Huh7 cells, and **(B)** the Western blot was utilized to examine the expression of *NAP1L1*, *β-catenin*, and *GAPDH*.

The aforementioned results show that the G2/M phase transition of the cell cycle and *Wnt* signaling pathway can be found in the GSE14520 dataset, GSE76427 dataset, ICGC dataset, and TCGA dataset through GSEA. Then, the *NAP1L1* expression level was measured in NC, si-1, si-2, and si-3 groups in MHCC-97H and Huh7 cell lines (Figure 6A) *via* the Western blot. The outcomes demonstrated no changed features in NC and si-3 groups, but si-1 and si-2 groups were evidently low in terms of *NAP1L1* expression levels in them.

*β-catenin* was regarded as a protein to regulate the *Wnt* signaling pathway. *CDK1* was regarded as a protein to regulate G2 to M phase transition of the cell cycle. The expression of *β-catenin* and *CDK1* was measured using the Western blot (Figure 6B, Supplementary Figure S4A). The outcomes showed that after *NAP1L1* silencing, *β-catenin* and *CDK1* both downregulate accordingly, which meant that the expression of *NAP1L1* had a positive relationship between *β-catenin* and *CDK1*. In addition, after *NAP1L1* silencing, cell numbers in the G2 phase (Supplementary Figures S4B, C) were significantly increased in both MHCC-97H and Huh7 cells, measured by the flow cytometry assay.

Therefore, we believed that *NAP1L1* knockdown could inhibit HCC cell proliferation by inhibiting *Wnt*/*β-catenin* pathway activation and the G2/M phase of cell cycle transition.

### 3.6 The Analysis of Immune Infiltration Ground on GSE14520 Dataset, GSE76427 Dataset, ICGC Dataset, and TCGA Dataset

**FIGURE 7:**
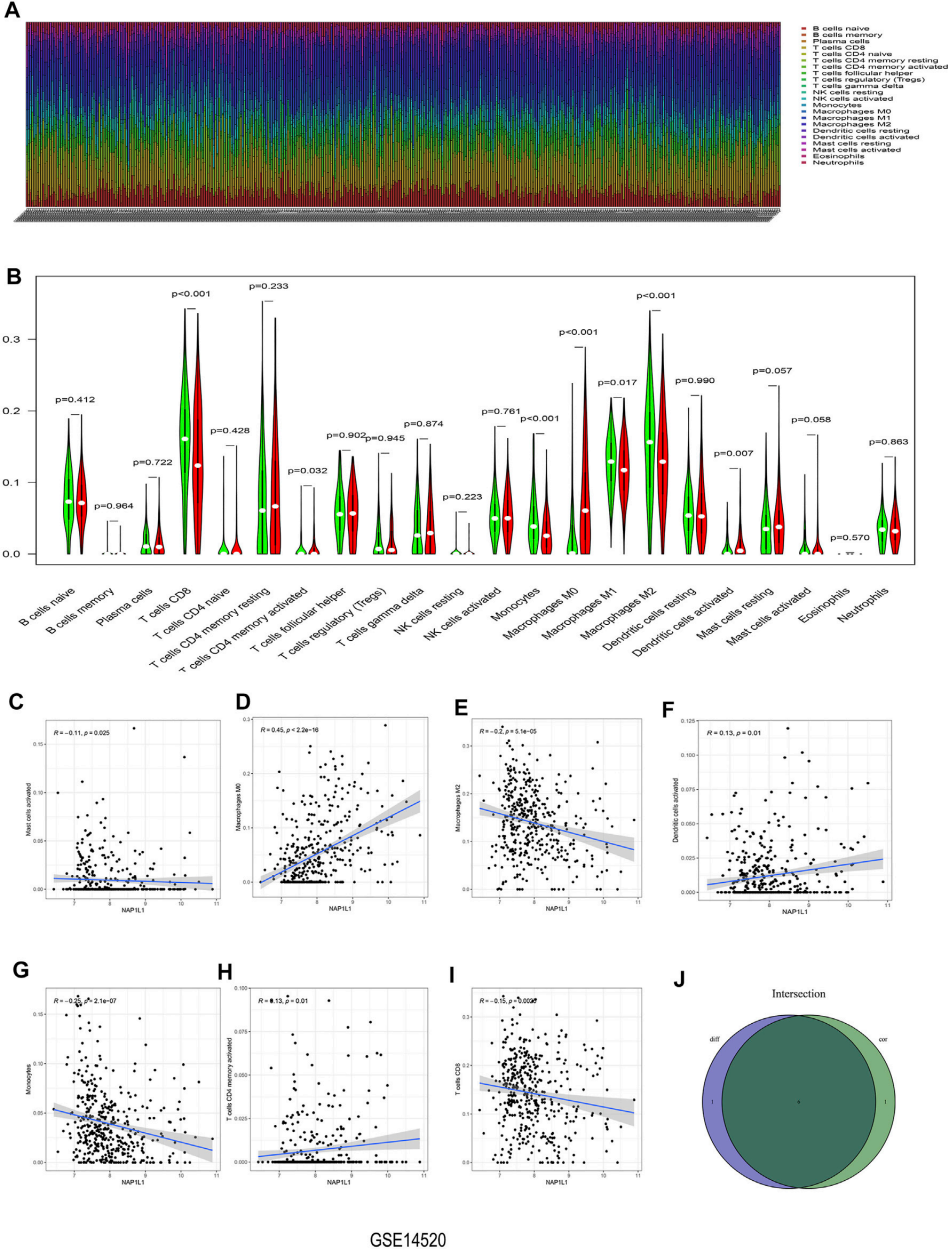
Analysis of immune infiltration in the GSE14520 dataset. **(A)** The bar graph containing the 22 immune cells. **(B)** The violin graph containing the 22 immune cells. **(C)** The relationship between *NAP1L1* and activated mast cells. **(D)** The relationship between *NAP1L1* and M0 macrophages. **(E)** The relationship between *NAP1L1* and M2 macrophages **(F)** The relationship between *NAP1L1* and activated dendritic cells. **(G)** The relationship between *NAP1L1* and monocytes. **(H)** The relationship between *NAP1L1* and activated memory CD4 T cells. **(I)** The relationship between *NAP1L1* and CD8 T cells. **(J)** The Venn graph was utilized to picture the meaningful immune cells between the violin plot and the correlation graph.

For exploring the association between *NAP1L1* and the immune cells in HCC patients, we conducted the following analysis:

The CIBERSORT algorithm was taken to acquire the landscape of tumor-infiltrating immune cells. Bar plots were painted through R studio to test the correlation between NAP1L1 expression and 22 immune cells. From those bar plots (Figure 7A, Supplementary Figures S5A, S6A, S7A), we can observe the percent of 22 immune cells in each HCC sample. The violin plot was utilized to recognize the variety of 22 immune cells between the high and the low expression groups in HCC samples ground on the median expression level of *NAP1L1*. Furthermore, from observing those violin plots (Figure 7B, Supplementary Figures S5B, S6B, S7B), we found a remarkable difference in activated memory CD4 T cells, activated dendritic cells, gamma delta T cells, regulatory T cells (Tregs), CD8 T cells, activated NK cells, monocytes, M0 macrophages, M1 macrophages, and M2 macrophages.

The correlation graph was utilized to recognize the correlation between *NAP1L1* and 22 immune cells. Then these significant immune cells were selected and presented in the following figures. They activated Mast cells (Figure 7C), M0 macrophages (Figure 7D, Supplementary Figures S5C, S7D), M2 macrophages (Figure 7E, Supplementary Figures S6E), activated dendritic cells (Figure 7F), monocytes (Figure 7G), activated memory CD4 T cells (Figure 7H), CD8 T cells (Figure 7I), M1 macrophages (Supplementary Figures S6D, S7E), gamma delta T cells (Supplementary Figure S5D), naive B cells (Supplementary Figure S6C), activated NK cells (Supplementary Figure S6F), and memory B cells (Supplementary Figure S7C). From those Venn graphs of meaningful immune cells in the violin plot and correlation graph, common immune cells were selected in GSE14520 datasets (Figure 7J), GSE76427 datasets (Supplementary Figures S5E), ICGC datasets (Supplementary Figure S6G), and TCGA datasets (Supplementary Figure S7F). We found CD8 T cells, activated memory CD4 T cells, monocytes, M0 macrophages, M2 macrophages, and activated dendritic cells as the common immune cells in GSE14520 dataset. M0 macrophages and gamma delta T cells were the common immune cells in GSE76427 datasets. Moreover, activated NK cells and M1 macrophages were common immune cells in ICGC dataset. Finally, M0 macrophages and M1 macrophages were the common immune cells in TCGA dataset.

In Conclusion, the aforementioned analysis of immune infiltration in GSE14520 dataset, GSE76427 dataset, ICGC dataset, and TCGA dataset showed that remarkable differences in immune cells existed in HCC patients, and the correlations between the expression of NAP1L1 and some immune cells were statistically significant. Finally, we speculated that NAP1L1 expression is most associated with macrophages in HCC.

### 3.7 The Analysis of Immune Infiltration Ground on TISIDB and TIMER Online Databases

**FIGURE 8:**
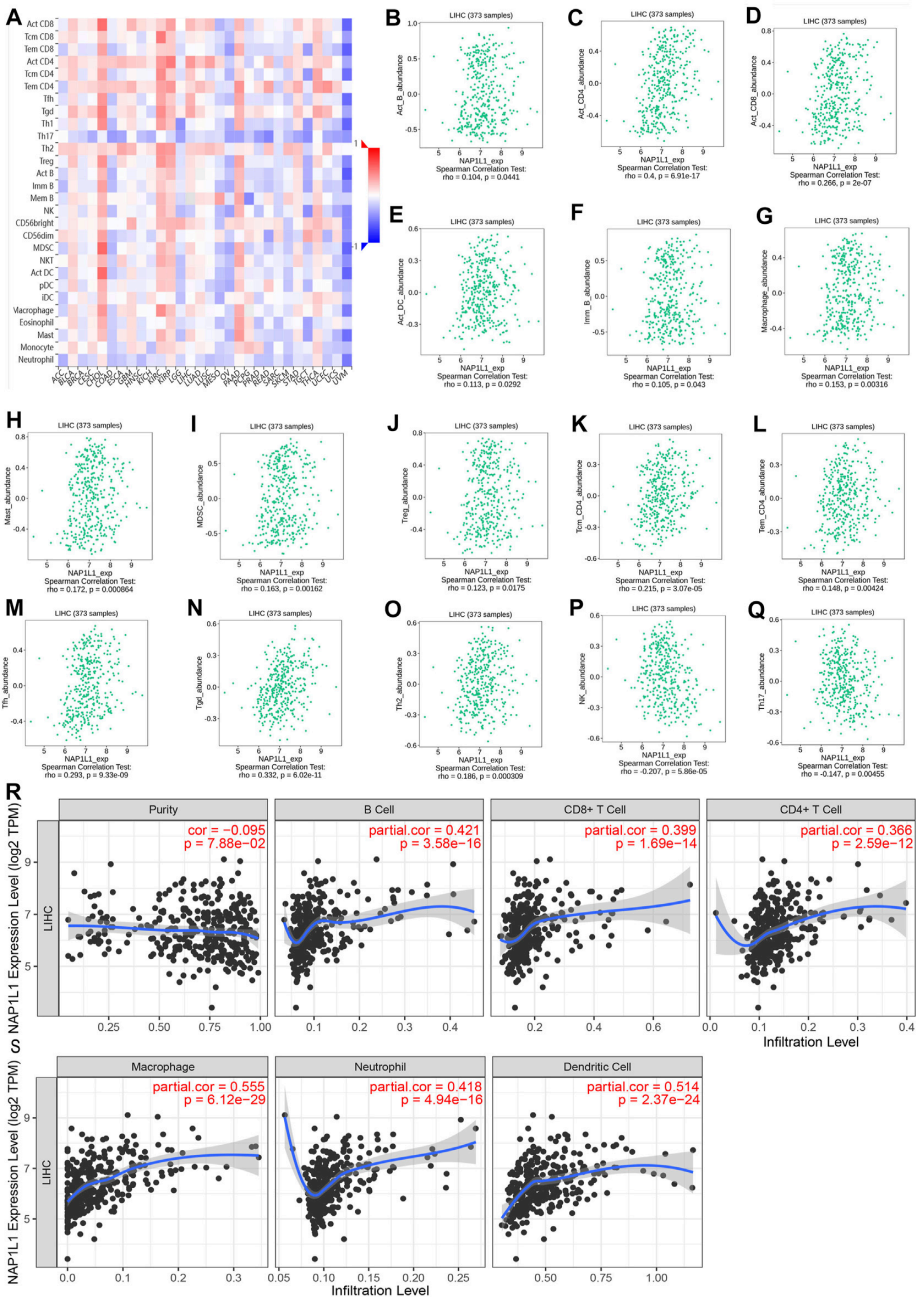
Analysis of immune infiltration in TISIDB and TIMER. **(A)** The panoramic association between the NAP1L1 expression and TILs in human cancer (red represents the positive correlation, and blue represents the negative correlation). **(B–O)** The NAP1*L1* expression was positively related to the infiltrating levels of the immune cell in HCC. **(P,Q)** The *NAP1L1* expression was negatively related to the infiltrating levels of the immune cell in HCC. **(R,S)** The *NAP1L1* expression is correlated with the level of immune infiltration.

The association between the enrichment of TILs and the expression of *NAP1L1* were inferred using the TISIDB database, and the correlation between the *NAP1L1* expression and the TILs of people’s cancer is displayed in Figure 8A. It was a certain association between the enrichment of 28 TIL types and the expression of *NAP1L1*. Specifically speaking, *NAP1L1* expression was closely positively related to the following immune cells: activated B cells (*r* = 0.104, *p* = 0.0441), activated CD4 T cells (*r* = 0.4, *p* = 0.6.9e-17), activated CD8 T cells (*r* = 0.266, *p* = 2e-07), activated dendritic cells (*r* = 0.104, *p* = 0.0292), immature B cells (*r* = 0.105, *p* = 0.0443), macrophage (*r* = 0.153, *p* = 0.00316), mast cells (*r* = 0.172, *p* = 0.000864), myeloid-derived suppressor cells (*r* = 0.163, *p* = 0.00162), regulatory T cells (*r* = 0.123, *p* = 0.0175), central memory CD4 T cells (*r* = 0.215, *p* = 3.07e-05), effector memory CD4 T cells (*r* = 0.148, *p* = 0.004), T follicular helper cells (*r* = 0.293, *p* = 9.33e-09), gamma delta T cells (*r* = 0.332, *p* = 6.02e-11), and Type-2 T helper cells (*r* = 0.186, *p* = 0.000309) (Figure 8B-O). In addition, the expression of *NAP1L1* was deeply negatively relevant to the following immune cells: natural killer cells (*r* = −c0.207, *p* = 5.86e-05) and Type-17 helper cells (*r* = −0.147, *p* = 0.00455) (Figures 8P, Q). Last, the TIMER database was utilized to further verify the relations between the *NAP1L1* expression and the immune cell infiltration in HCC (Figures 8R, S). The outcomes displayed that the *NAP1L1* expression was closely relevant to the following immune cells: B cells (*p* = 3.58e-16), CD8^+^ T cells (*p* = 1.69e–14), CD4^+^ T cells (*p* = 2.59e–12), macrophages (*p* = 6.12e–29), neutrophils (*p* = 4.94e–16), and dendritic cells (*p* = 2.37e–24).

To summarize, through the comprehensive analysis of GSE14520, GSE76427, ICGC, TCGA, TISIDB, and TIMER databases, we had a hypothesis that *NAP1L1* expression was related to immune cell and *NAP1L1* may also accelerate the HCC progression by affecting macrophage through some underlying mechanisms.

## 4 Discussion

Hepatocellular carcinoma (HCC) is regarded as one of the universal cancers worldwide. As for the world cancer data in 2020, the incidence of HCC ranked the seventh, and the mortality rate related to HCC ranked the fourth in the world (Sung et al., 2021). Although the systemic treatment of HCC has made a significant progress in these years, the extreme unbalance of survival and recurrence of HCC patients is still a significant difficulty. It is an imperious topic to be solved in HCC today. Therefore, it is an urgent need to research the association between the hub gene and the occurrence, progression, or prognosis in HCC patients. This study may lead us to acquire some achievements in genes or diseases that the biomarker, the whole mechanisms, and a novel treatment method in HCC patients can be further promoted in the current and future.

Therefore, we screened out 2,145 DEGs and 101 survival-related genes through bioinformatics analysis from four datasets (GSE14520, GSE76427, ICGC, and TCGA) about HCC 33 common genes among them. Last, the 33 common genes were applied to conduct a PPI network *via* String. The top 10 key genes were chosen utilizing 12 different methods by the cytoHubba plug-in of Cystoscope *via* exploring the 12 outcomes, so we chose our hub gene, *NAP1L1*.

*NAP1L1* is a critical member of the Nucleosome Assembly Protein (NAP) family and has been widely portrayed in various tumor progressions (Aydin et al., 2020; Chen et al., 2021; Liu X. et al., 2021). The high expression of *NAP1L1* has existed in the heap of human neoplasms covering nasopharyngeal carcinoma (Liu S. et al., 2021), lung adenocarcinoma (Nagashio et al., 2020), glioblastoma (Zottel et al., 2020), colon cancer (Aydin et al., 2020), colorectal cancer (Queiroz et al., 2020), breast cancer (Liu S. et al., 2021), small-intestinal carcinoid (Kidd et al., 2006), and pancreatic neuroendocrine neoplasm (Schimmack et al., 2014). In addition, previous studies have confirmed the high expression was relevant to the worse survival in nasopharyngeal carcinoma ( Liu X. et al., 2021), lung adenocarcinoma (Nagashio et al., 2020), colon cancer (Aydin et al., 2020), and breast cancer (Liu S. et al., 2021). However, we only found seldom studies of HCC. First, the high expression of *lncRNA CDKN2B-AS1* could accelerate the development of HCC regulating the *PI3K*/*AKT*/*mTOR* signaling pathway in which *NAP1L1* was a target of *lncRNA CDKN2B-AS1* (Huang et al., 2018). In addition, the *PI3K*/*AKT*/*mTOR* signaling pathway was inhibited in *PRDM8* overexpression by the regulation of *NAP1L1* in HCC, which made an antitumor effect (Chen Z. et al., 2018). The upregulation of *NAP1L1* can recruit *HDGF*/*c-Ju*n, boosting the HCC progression (Zhang et al., 2021). Other scholars claimed that the proliferation of HCC cells and chemotherapy resistance could be enhanced after the high expression of *NAP1L1* (Le et al., 2019). The aforementioned studies showed that *NAP1L1* could promote HCC progression *via* activating the *PI3K*/*AKT*/*mTOR* signaling pathway and recruiting *HDGF*/*c-Jun*. Meanwhile, we observed that *NAP1L1* could promote HCC progression through other mechanisms, so the study series was conducted in the following.

On the one hand, survival analysis, clinical feature analysis, univariate/multivariate regression analysis, and GSEA were used to study *NAP1L1* by collecting HCC patients from GSE14520, GSE76427, ICGC, and TCGA. The Conclusion presented that HCC patients with higher expression levels of *NAP1L1* had a poorer prognosis than those with lower expression levels, and *NAP1L1* is an independent prognostic factor for HCC. Although the relation between the expression of *NAP1L1* and the HCC patients’ survival and prognostic had been studied, the HCC progression is vague today. In order to shed light on NAP1L1’s molecular mechanism promoting the progression of HCC closely, GSEA was applied in GSE14520, GSE76427, ICGC, and TCGA. The results confirmed that *NAP1L1* could promote HCC progression by regulating the G2/M transition of the cell cycle and the *Wnt* signaling pathway. Thus, the hypothesis of HCC progression influenced by *NAP1L1* may be achieved by regulating the G2/M phase and activating the *Wnt*/*β-catenin* pathway.

On the other hand, the Western blot and the flow cytometry experiments were utilized to prove our summary. The result of the Western blot showed that *NAP1L1* silencing led to downregulation of *CDK1* and *β-catenin* proteins; the result of flow cytometry was that cell numbers in the G2 phase were significantly increased when *NAP1L1* silenced. Some scholars thought that *CDK1* was gathered in the G2 phase of the cell cycle during the late period and *CDK1* was required to regulate it (Bonelli et al., 2014; Roskoski, 2019). Another study has also found that promoting the degradation of *CDK1* can block the G2/M transition of the cell cycle (Lee et al., 2016). Then, the *Wnt*/*β-catenin* pathway is mainly inactive in a healthy liver. However, it is frequently activated and promotes tumor growth in HCC. It strictly gets command of the embryogenesis, covering hepatobiliary development, maturation, and zonation (Perugorria et al., 2019). Many previous studies have confirmed that *Wnt*/*β-catenin* pathway activation could accelerate the progression of HCC (Huang et al., 2020; Tian et al., 2020). Our previous experimental results showed that downregulation of CDK1, β-catenin proteins, and cell numbers in the G2 phase significantly increased when NAP1L1 silences. Therefore, we speculate that upregulation of *NAP1L1* may boost the HCC progression by promoting *Wnt*/*β-catenin* signaling pathway activation and G2/M phase transition of the cell cycle.

The previous studies have also thought that activating the *Wnt*/*β-catenin* signaling pathway can promote M2 polarization in macrophages (Feng et al., 2018; Tian et al., 2020). Therefore, ground on GSE14520 and GSE76427 datasets, ICGC and TCGA databases, and TISIDB and TIMER databases, the correlation between NAP1L1 and immune cells was analyzed by applying HCC patients. To sum up, *NAP1L1* expression related to immune cells and *NAP1L1* may also accelerate the HCC progression by affecting macrophages through some underlying mechanisms. Nonetheless, the results are based on the bioinformatics analysis, which needs more exploration and research in the following study. One study has found that the TAM was the immunosuppressive cell, functioned as a kind of suppression antitumor immunity, and promoted the progression of the tumor via expressing the cytokines and chemokines. The TAM is a crucial component in promoting HCC growth and invasion of the tumor microenvironment (Lu et al., 2019). The other study has shown the inhibition of the TAM infiltration. Furthermore, the macrophage M2 polarization could reverse the immunosuppressive state of the tumor microenvironment and activate the responses of the antitumor CD8+ T cells (Li X. et al., 2017). The activation of the *Wnt*/*β-catenin* pathway could accelerate the macrophage M2 polarization (Yang et al., 2018), so inhibiting the *Wnt*/*β-catenin* pathway may be a potential strategy for treating HCC patients. We confirmed that high expression of *NAP1L1* can stimulate the *Wnt*/*β-catenin* pathway to promote HCC progression. Therefore, the study of *NAP1L1* provides some novel thoughts and perspectives for the precise treatment of HCC.

According to the analysis of multiple databases about *NAP1L1*, the Conclusion showed that HCC patients with higher expression levels of NAP1L1 had a poorer prognosis than those with lower expression levels, and *NAP1L1* is an independent prognostic factor for HCC. The study further found that the hypothesis of HCC progression influenced by *NAP1L1* may be achieved by regulating the G2/M phase and activating the *Wnt*/*β-catenin* signaling pathway. What is more, *NAP1L1* expression related to immune cells and *NAP1L1* also may accelerate the HCC progression by affecting macrophages through some underlying mechanisms. Nonetheless, the results are based on the bioinformatics analysis, which needs more exploration and research in the subsequent study.

## 5 Conclusion

Through the comprehensive analysis of four datasets, we figured out the hub gene (*NAP1L1*) in which the higher expression of *NAP1L1* was connected with HCC patients’ shorter survival time and poorer prognosis. *NAP1L1* is an independent prognostic factor in HCC. In addition, the analysis of multiple databases and experiments were carried out to explore the *NAP1L1*-relevant mechanism. The result of GSEA showed that *NAP1L1* might accelerate the progression of HCC, which relied on the cell cycle, especially the G2/M phase transition and Wnt signaling pathway. The result of the Western blot showed that *NAP1L1* silencing led to downregulation of *CDK1* and *β-catenin* proteins; the result of flow cytometry showed that cell numbers in the G2 phase were significantly increased when *NAP1L1* was silenced. Thus, we claimed that *NAP1L1* might promote HCC progression by activating the *Wnt* signaling pathway and promoting cell cycle G2/M transition. In the end, the correlation between *NAP1L1* and immune cells was analyzed by applying HCC patients. Furthermore, we also found that *NAP1L1* expression related to immune cells and *NAP1L1* may accelerate HCC progression by affecting macrophages through some underlying mechanisms. Nonetheless, the outcomes in the research are just based on the bioinformatics analysis, which needs more exploration and research in the future.

## Data Availability

The original contributions presented in the study are included in the article/Supplementary Material, further inquiries can be directed to the corresponding author.

## Abbreviations

*NAP1L1*, nucleosome assembly protein 1-like one; HCC, hepatocellular carcinoma; GEO, Gene Expression Omnibus; ICGC, International Cancer Genome Consortium Database; TCGA, The Cancer Genome Atlas; FPKM, the fragments per kilobase million; STRING, The Search Tool for the Retrieval of Interacting Genes; TIMER, The Tumor Immune Estimation Resource; TCIA, The Cancer Immunome Atlas; TISIDB, Tumor-immune System Interactions Database; DEGs, differentially expressed genes; PPI, protein–protein Interactions; KEGG, Kyoto Encyclopedia of Genes and Genomes; GSEA, Gene Set Enrichment Analysis; IPS, immuno pheno score; qRT-PCR; quantitative real-time PCR; DMEM, Dulbecco’s modified eagle medium; FBS, fetal bovine serum; OS, overall survival; HPA, Human Protein Atlas; TILs, tumor-infiltrating lymphocyte; NC, negative control; *PRDM8*, PRDI-BF1 and RIZ Homology Domain Containing eight; TAMs, tumor-associated macrophages.
