# Supplementary figures and images for: NAP1L1 Functions as a Novel Prognostic Biomarker Associated With Macrophages and Promotes Tumor Progression by Influencing the Wnt/β-Catenin Pathway in Hepatocellular Carcinoma
Source: Front Genet. 2022 May 19;13:876253. doi: 10.3389/fgene.2022.876253 (PMC9161088; doi:10.3389/fgene.2022.876253)

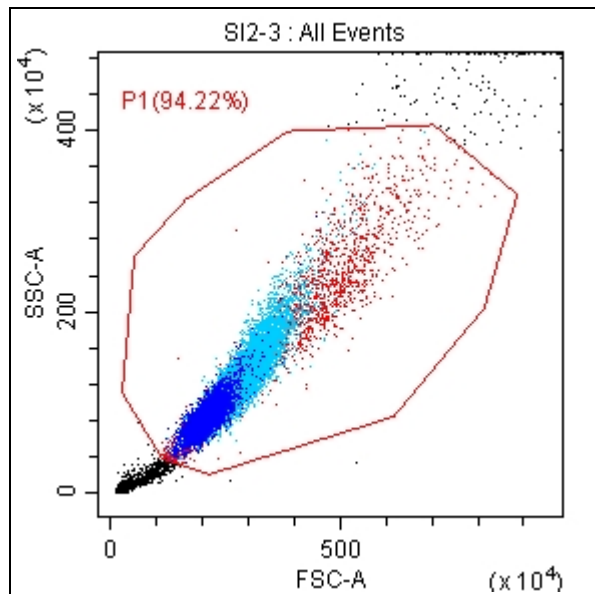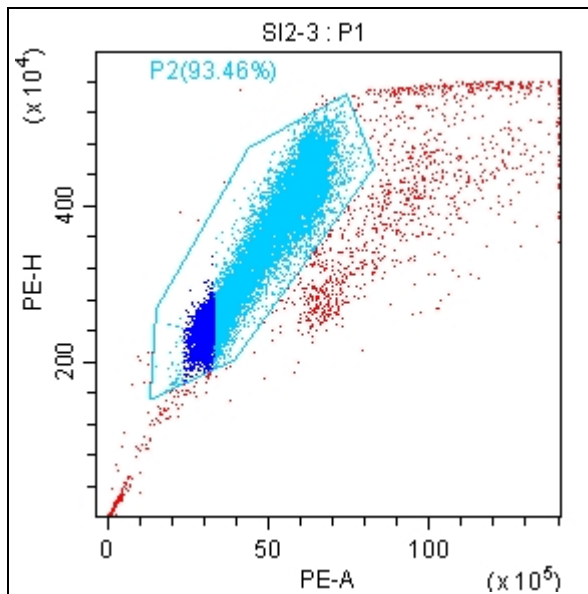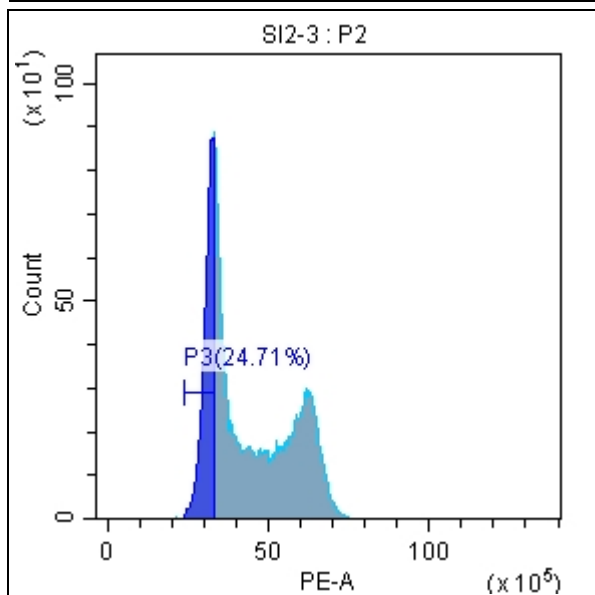

试管名称: SI2-3

样本ID:

| 群体           | 颗粒数   | %总数     | %父群     |
|--------------|-------|---------|---------|
| ● All Events | 22713 | 100.00% | 100.00% |
| ● P1         | 21400 | 94.22%  | 94.22%  |
| ● P2         | 20000 | 88.06%  | 93.46%  |
| ● P3         | 4941  | 21.75%  | 24.71%  |

Supplement: Supplementary file 1 [file DataSheet1.ZIP › origin data/flow cytometry/flow cytometry data/97H/NC.pdf]

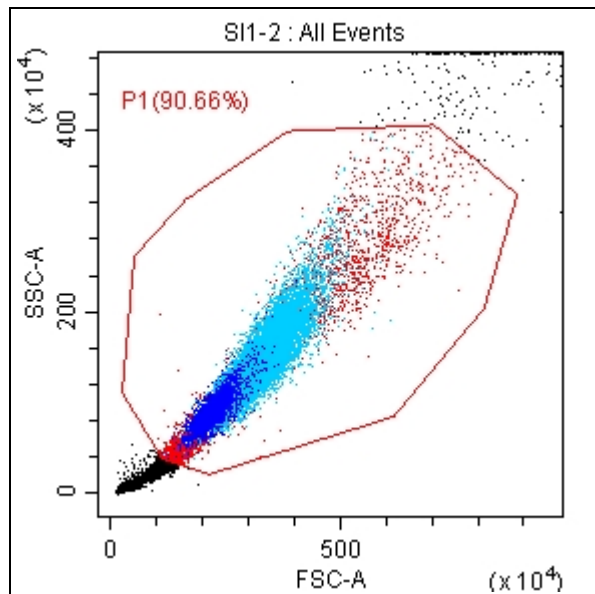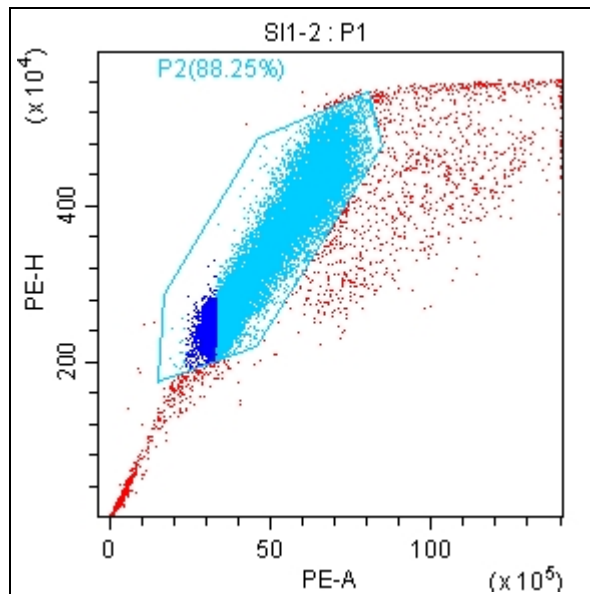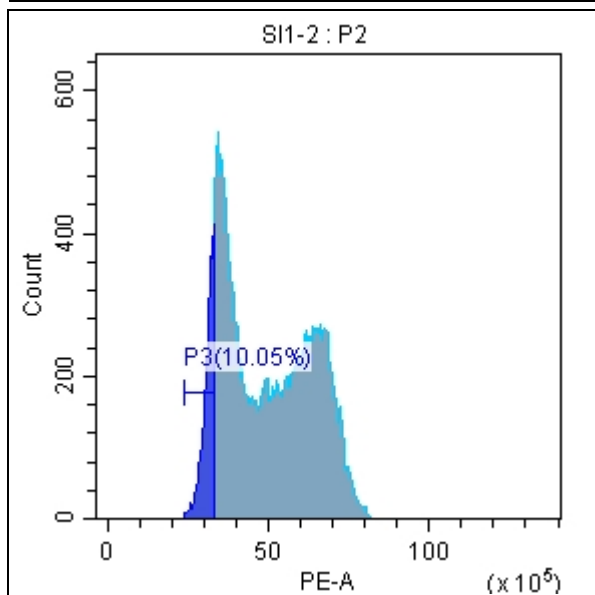

试管名称: SI1-2

样本ID:

| 群体           | 颗粒数   | %总数     | %父群     |
|--------------|-------|---------|---------|
| ● All Events | 24999 | 100.00% | 100.00% |
| ● P1         | 22664 | 90.66%  | 90.66%  |
| ● P2         | 20000 | 80.00%  | 88.25%  |
| ● P3         | 2009  | 8.04%   | 10.05%  |

Supplement: Supplementary file 1 [file DataSheet1.ZIP › origin data/flow cytometry/flow cytometry data/97H/Si1.pdf]

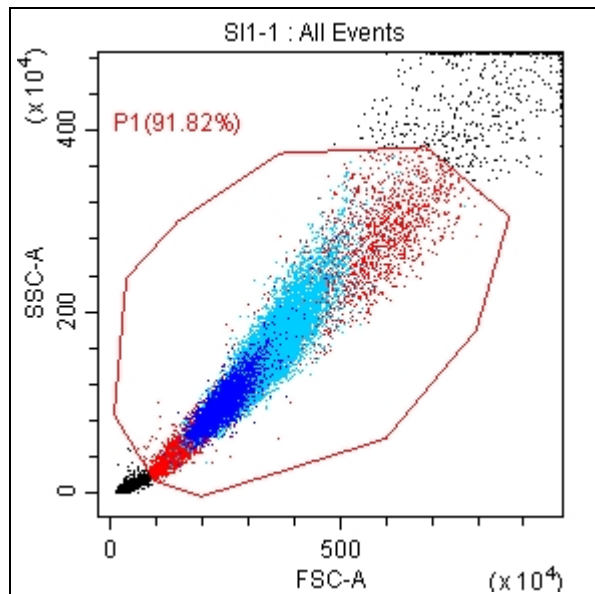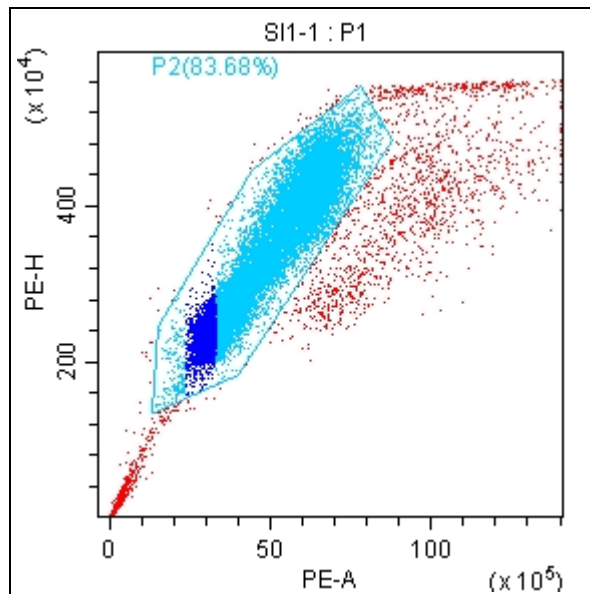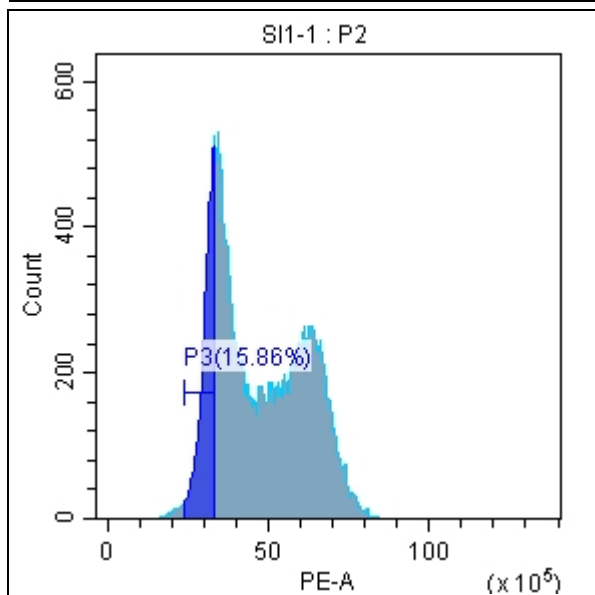

试管名称: SI1-1

样本ID:

| 群体           | 颗粒数   | %总数     | %父群     |
|--------------|-------|---------|---------|
| ● All Events | 26030 | 100.00% | 100.00% |
| ● P1         | 23900 | 91.82%  | 91.82%  |
| ● P2         | 20000 | 76.83%  | 83.68%  |
| ● P3         | 3171  | 12.18%  | 15.86%  |

Supplement: Supplementary file 1 [file DataSheet1.ZIP › origin data/flow cytometry/flow cytometry data/97H/Si2.pdf]

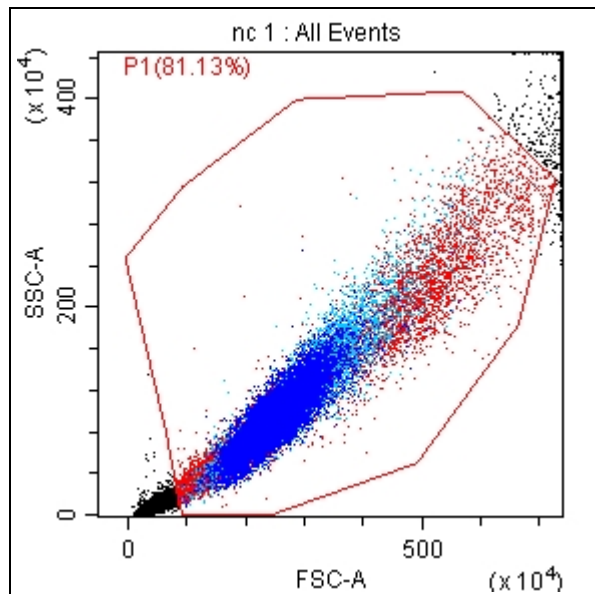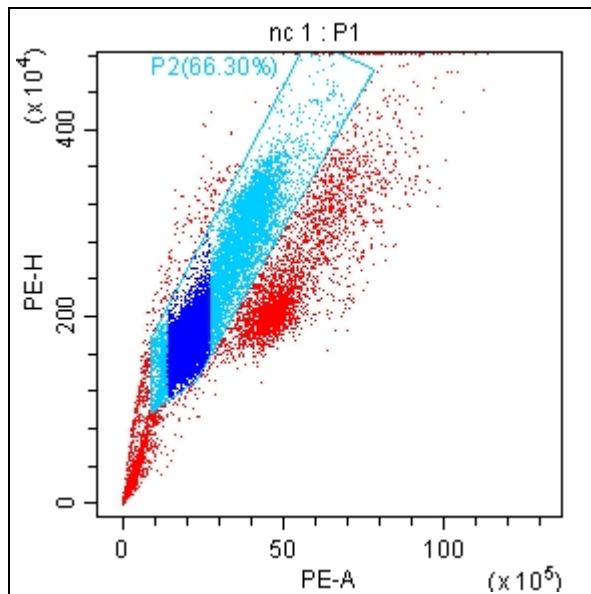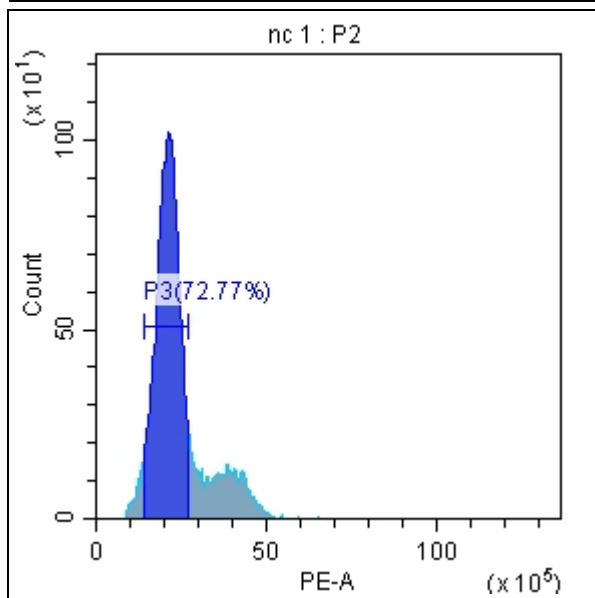

试管名称: nc 1

样本ID:

| 群体         | 颗粒数   | %总数     | %父群     |
|------------|-------|---------|---------|
| All Events | 38859 | 100.00% | 100.00% |
| P1         | 31526 | 81.13%  | 81.13%  |
| P2         | 20901 | 53.79%  | 66.30%  |
| P3         | 15209 | 39.14%  | 72.77%  |

Supplement: Supplementary file 1 [file DataSheet1.ZIP › origin data/flow cytometry/flow cytometry data/Huh7/NC.pdf]

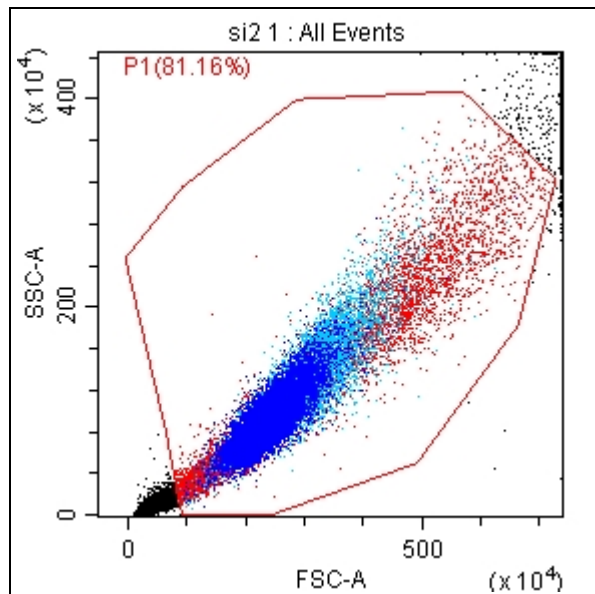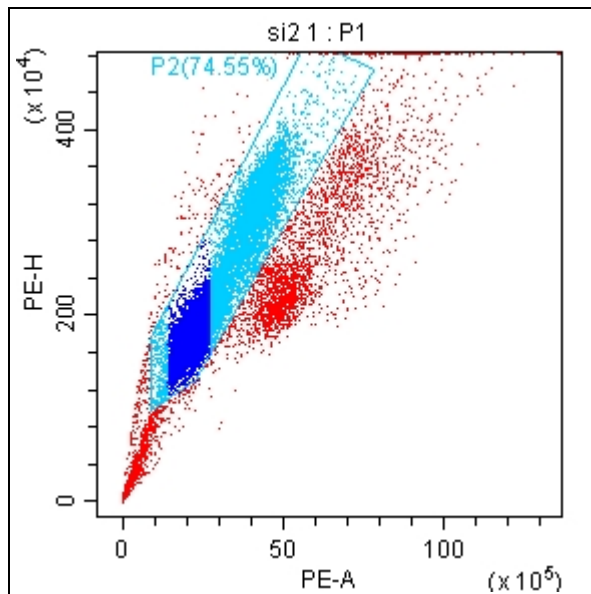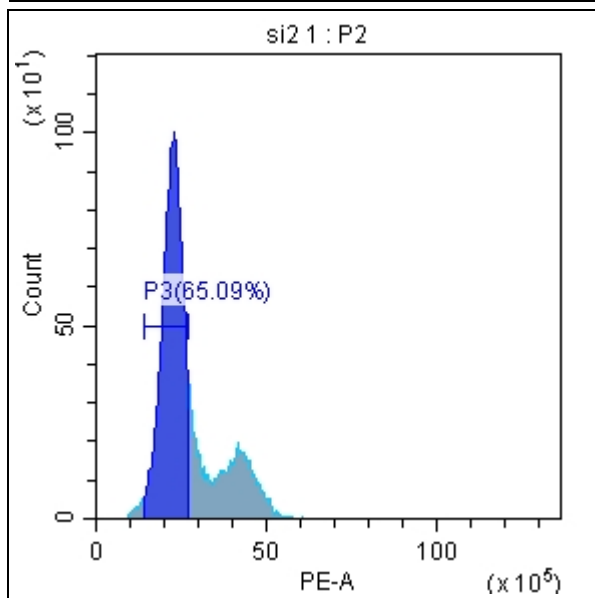

试管名称: si2 1

样本ID:

| 群体         | 颗粒数   | %总数     | %父群     |
|------------|-------|---------|---------|
| All Events | 33055 | 100.00% | 100.00% |
| P1         | 26829 | 81.16%  | 81.16%  |
| P2         | 20000 | 60.51%  | 74.55%  |
| P3         | 13018 | 39.38%  | 65.09%  |

Supplement: Supplementary file 1 [file DataSheet1.ZIP › origin data/flow cytometry/flow cytometry data/Huh7/Si1.pdf]

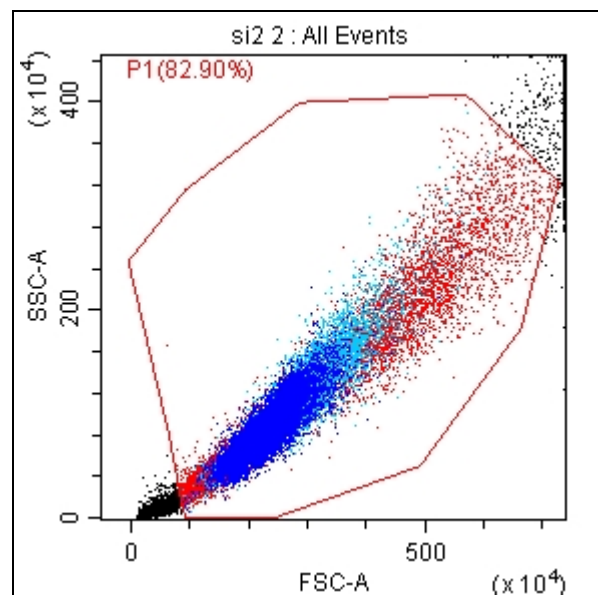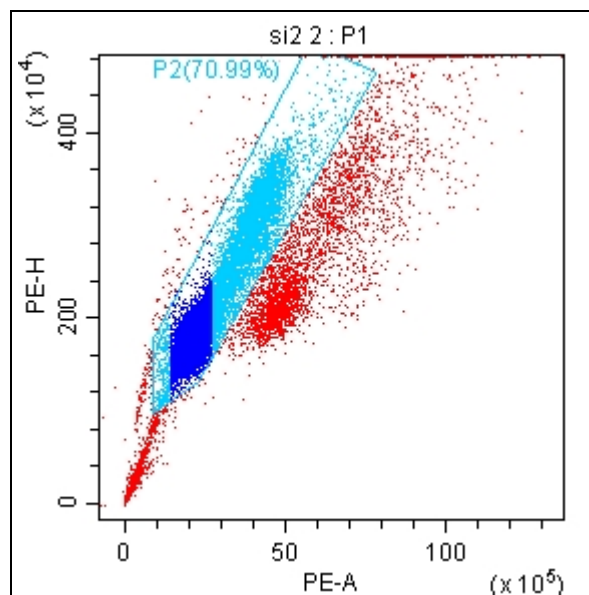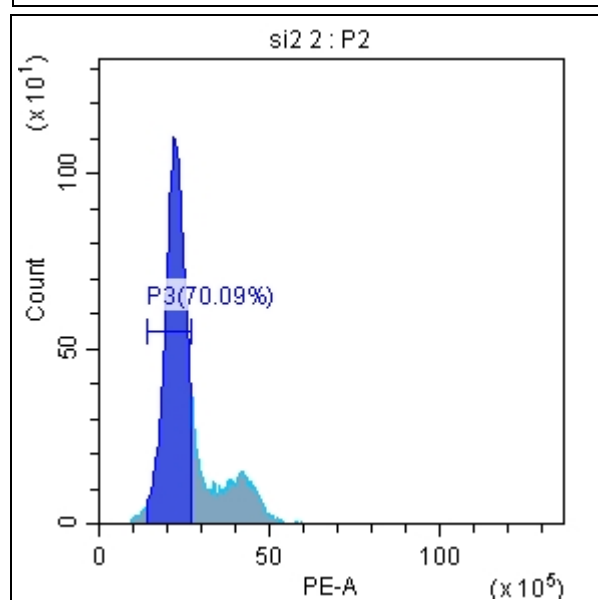

试管名称: si2 2

样本ID:

| 群体         | 颗粒数   | %总数     | %父群     |
|------------|-------|---------|---------|
| All Events | 33984 | 100.00% | 100.00% |
| P1         | 28172 | 82.90%  | 82.90%  |
| P2         | 20000 | 58.85%  | 70.99%  |
| P3         | 14018 | 41.25%  | 70.09%  |

Supplement: Supplementary file 1 [file DataSheet1.ZIP › origin data/flow cytometry/flow cytometry data/Huh7/Si2.pdf]

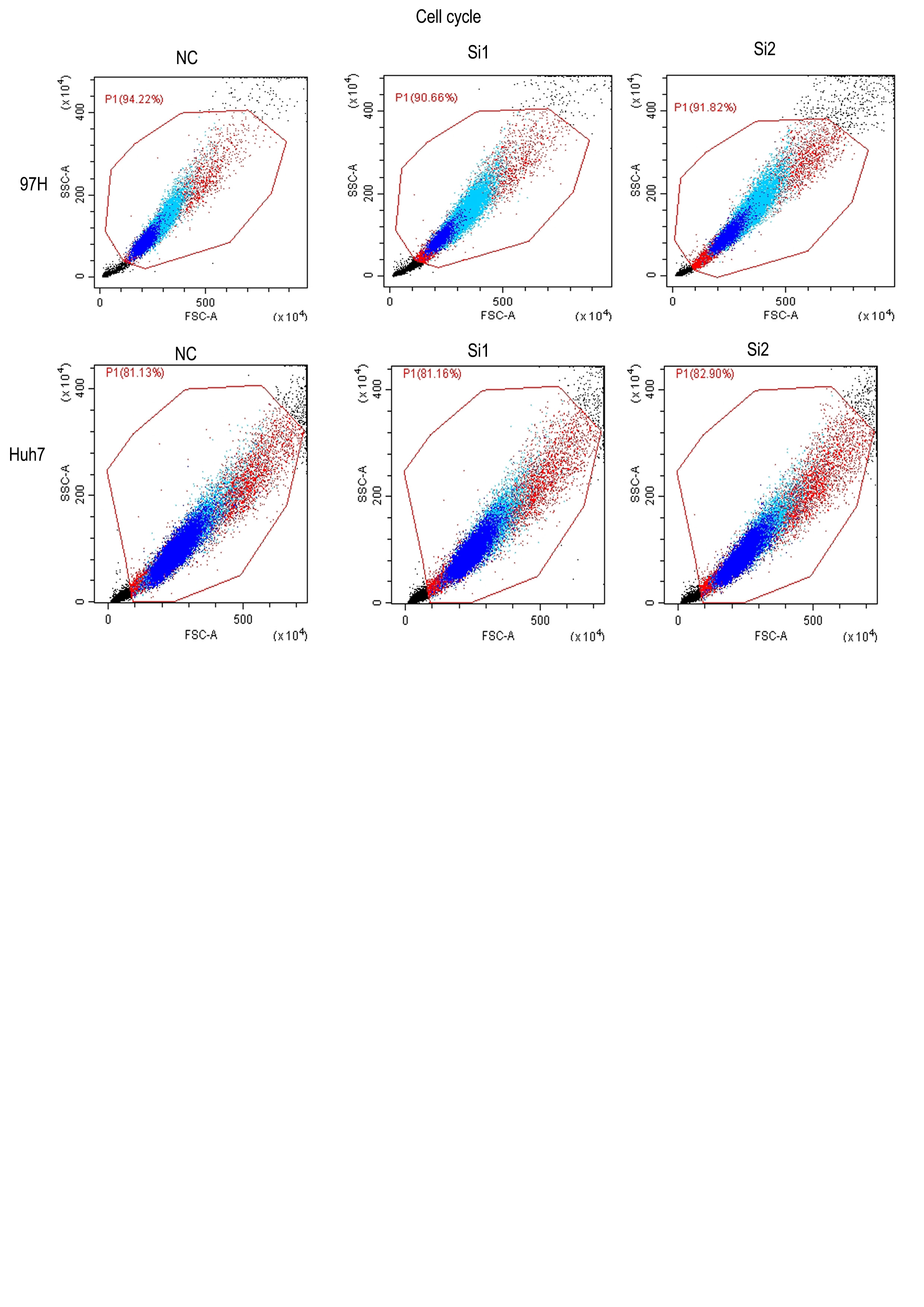

Supplement: Supplementary file 1 [file DataSheet1.ZIP › origin data/flow cytometry/gating strategies/gating strategies.jpg]

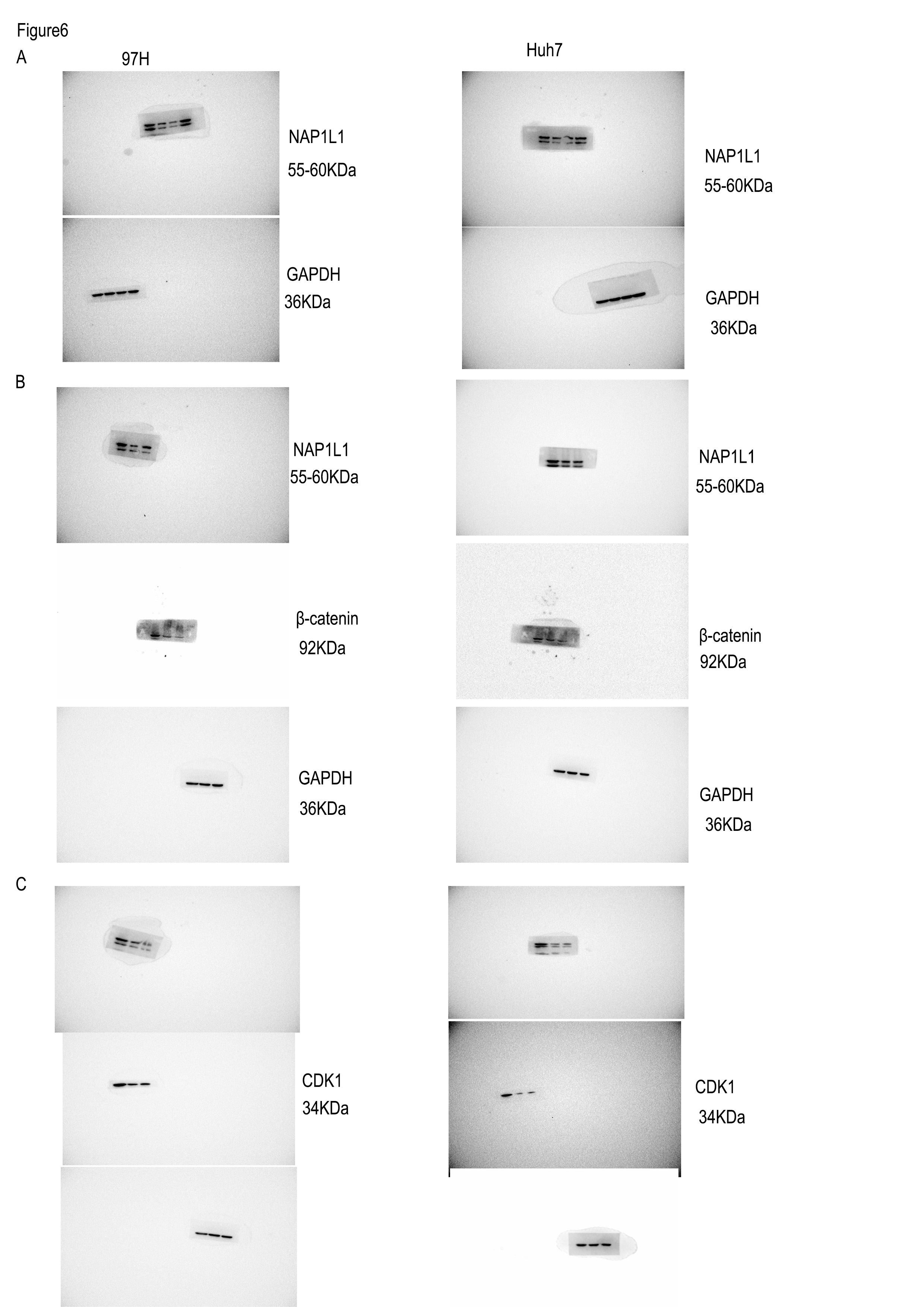

Supplement: Supplementary file 1 [file DataSheet1.ZIP › origin data/Western blot/WB of Figure2 and Figure8.jpg]

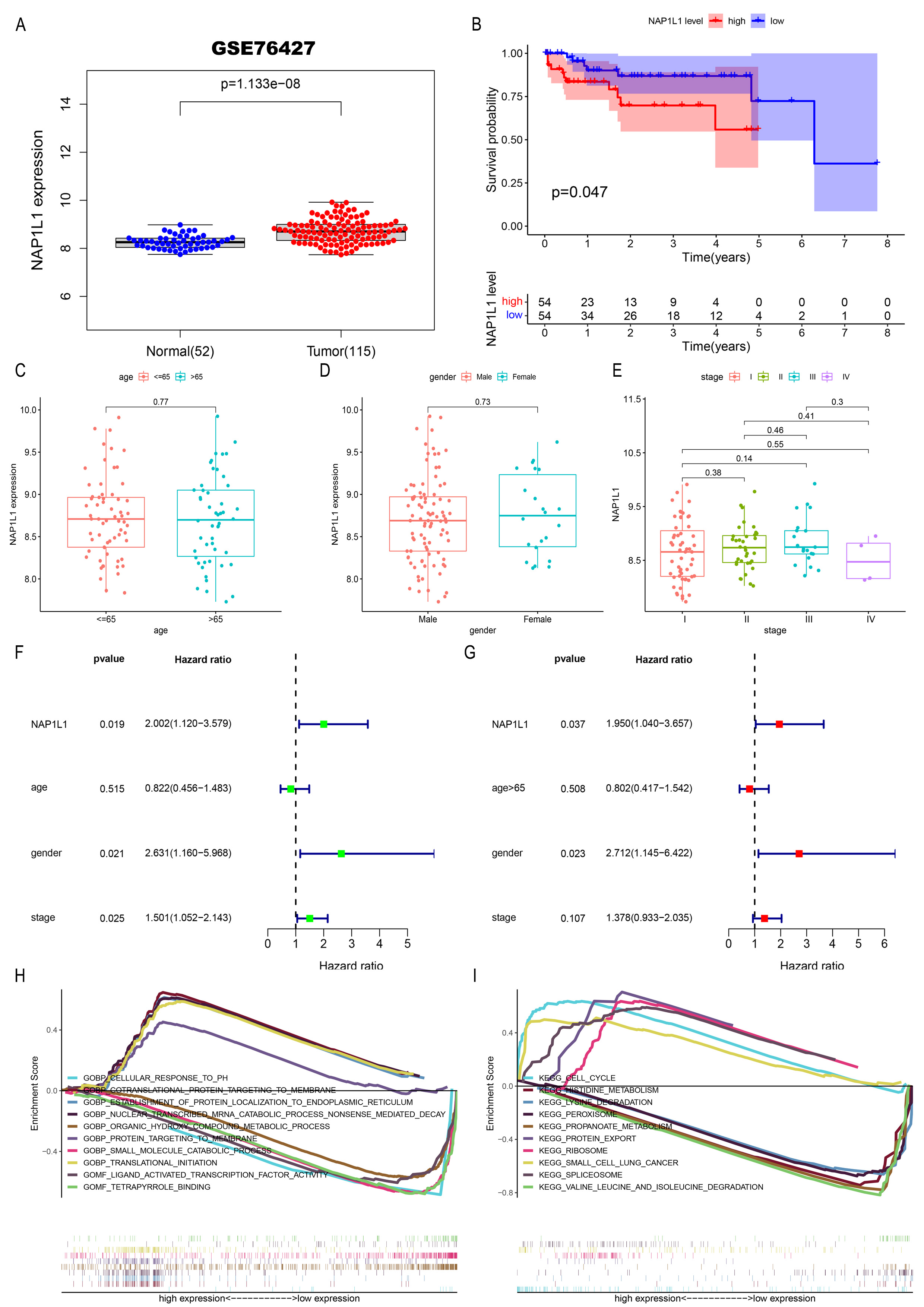

Supplement: Supplementary file 2 [file DataSheet2.ZIP › supplementary figure/Supplement Figure 1.jpg]

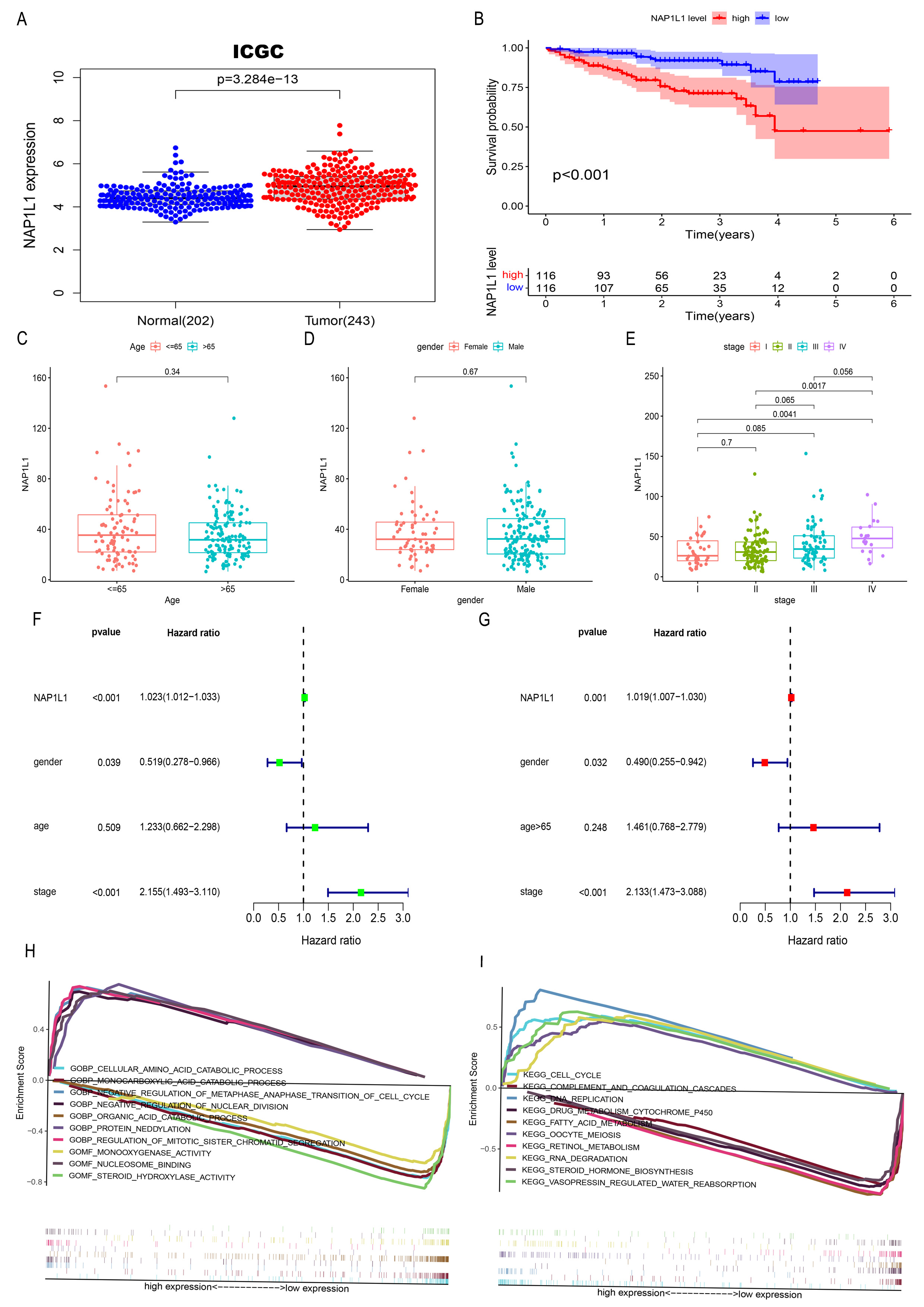

Supplement: Supplementary file 2 [file DataSheet2.ZIP › supplementary figure/Supplement Figure 2.jpg]

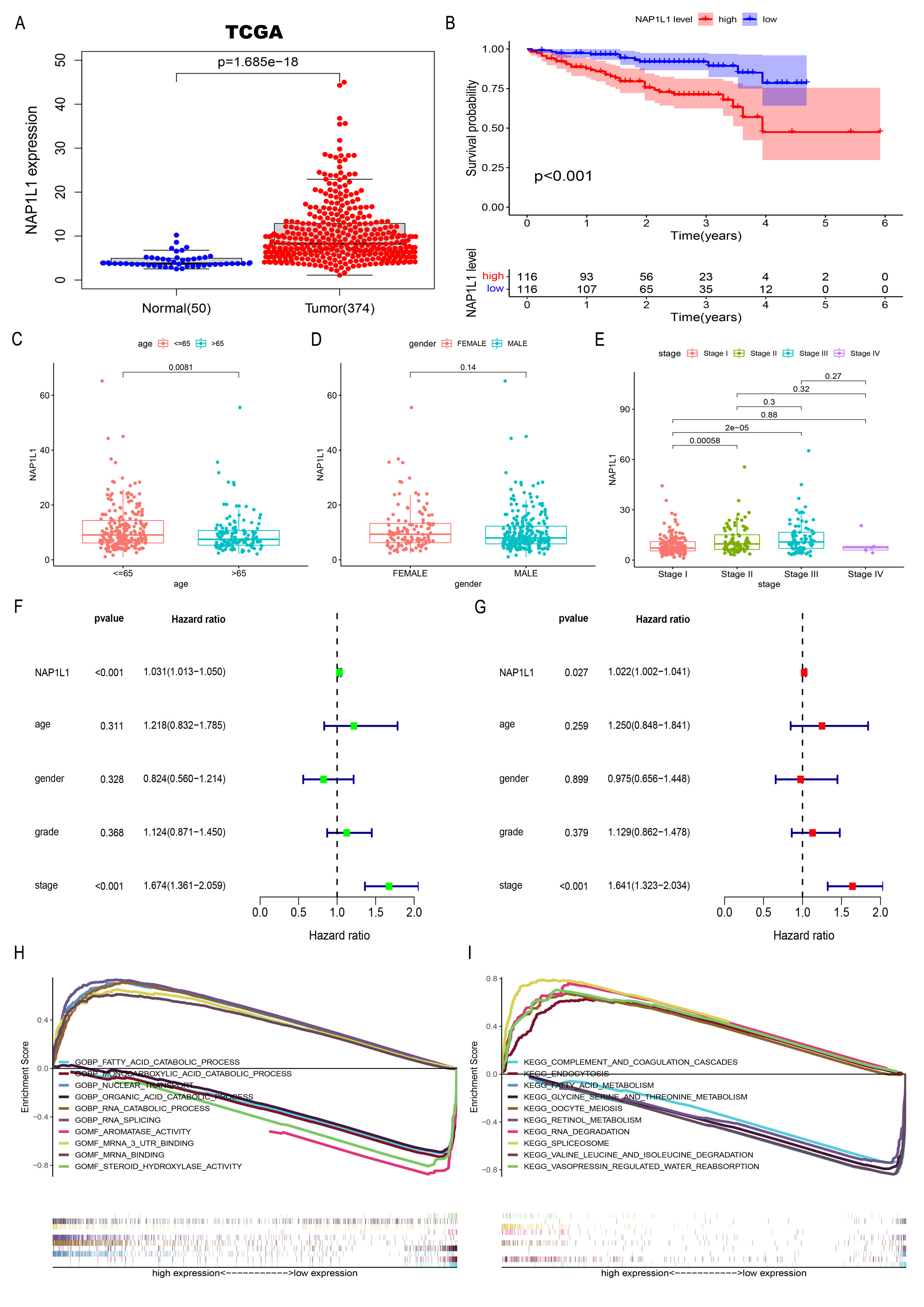

Supplement: Supplementary file 2 [file DataSheet2.ZIP › supplementary figure/Supplement Figure 3.jpg]

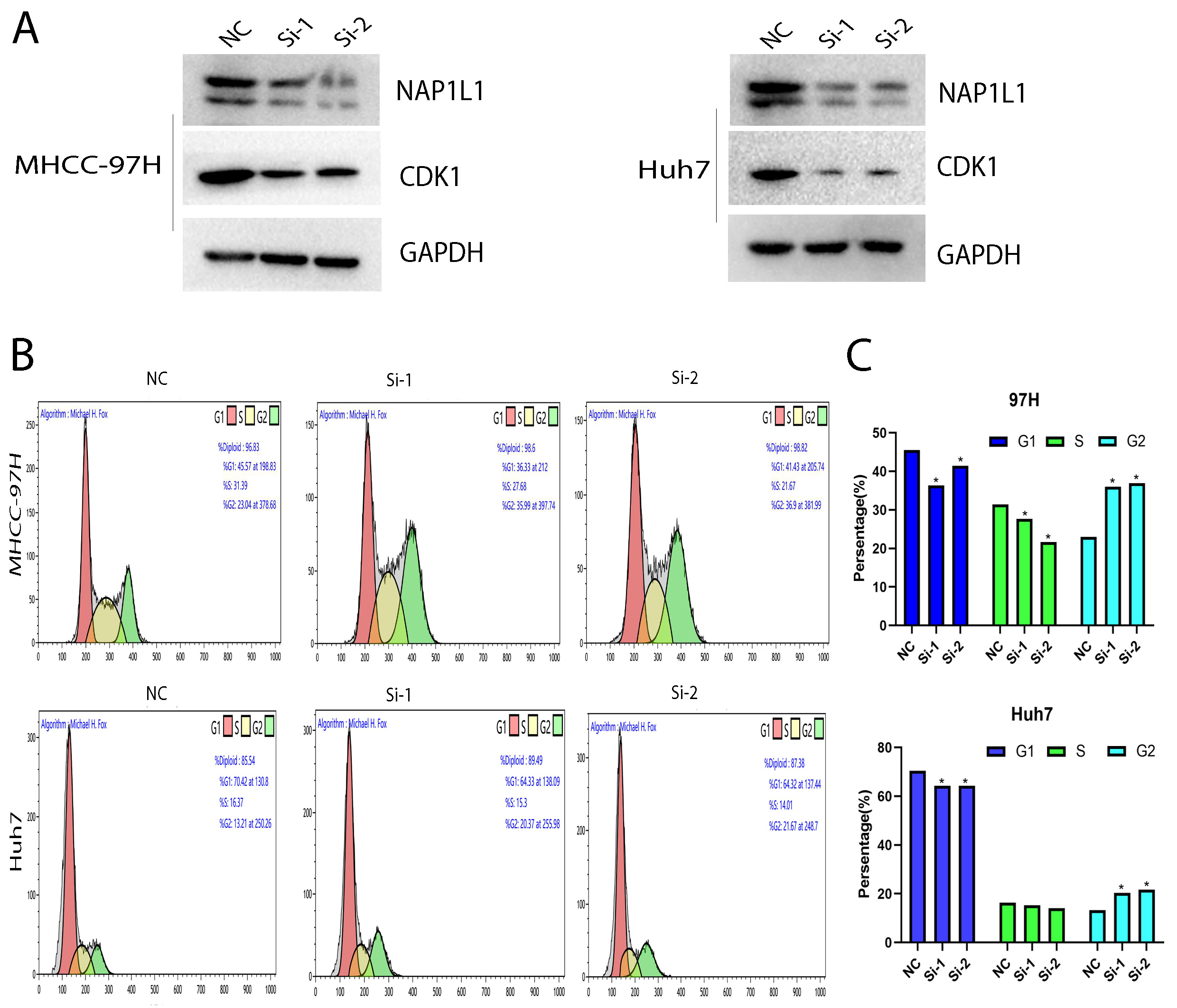

Supplement: Supplementary file 2 [file DataSheet2.ZIP › supplementary figure/Supplement Figure 4.jpg]

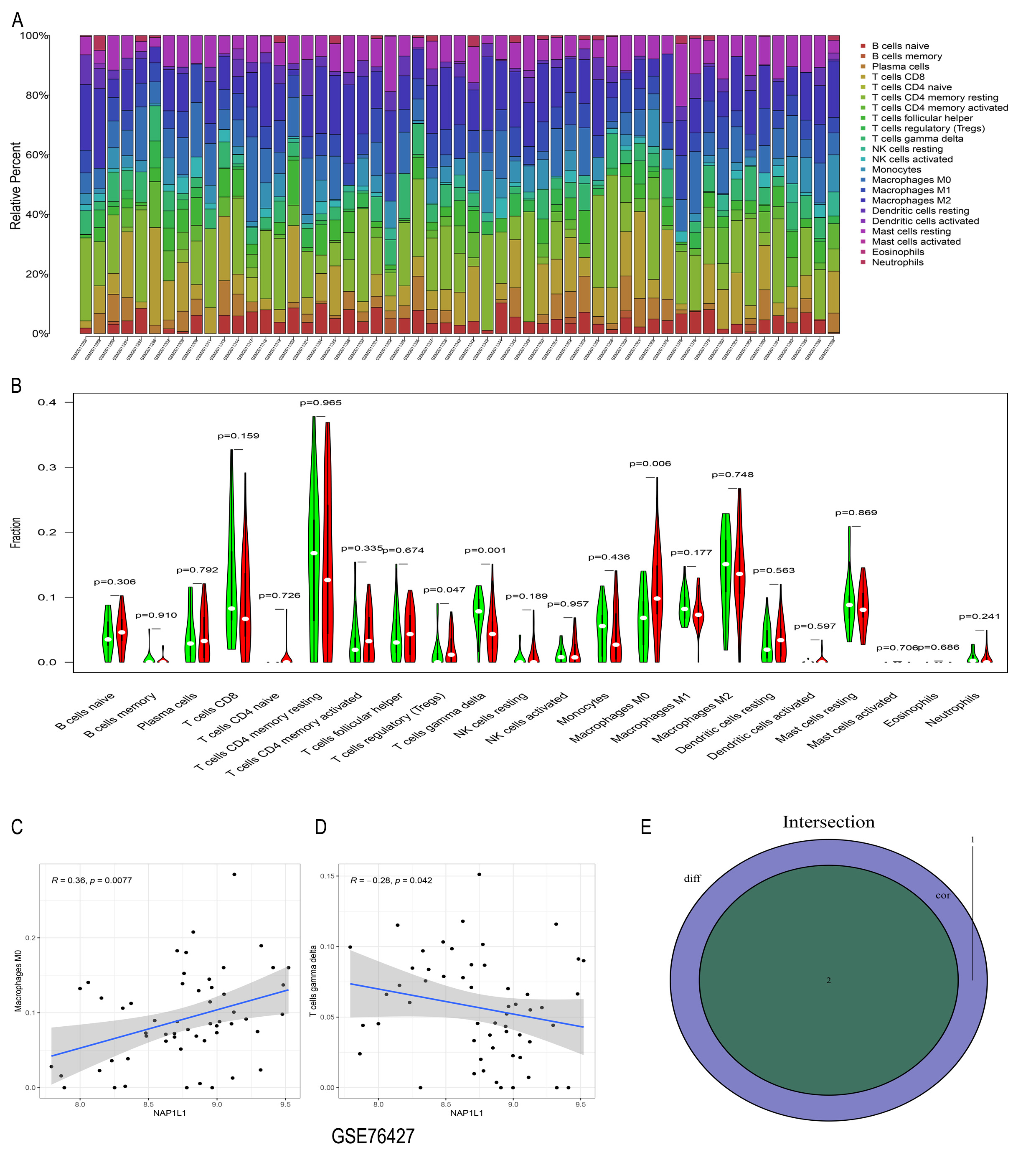

Supplement: Supplementary file 2 [file DataSheet2.ZIP › supplementary figure/Supplement Figure 5.jpg]

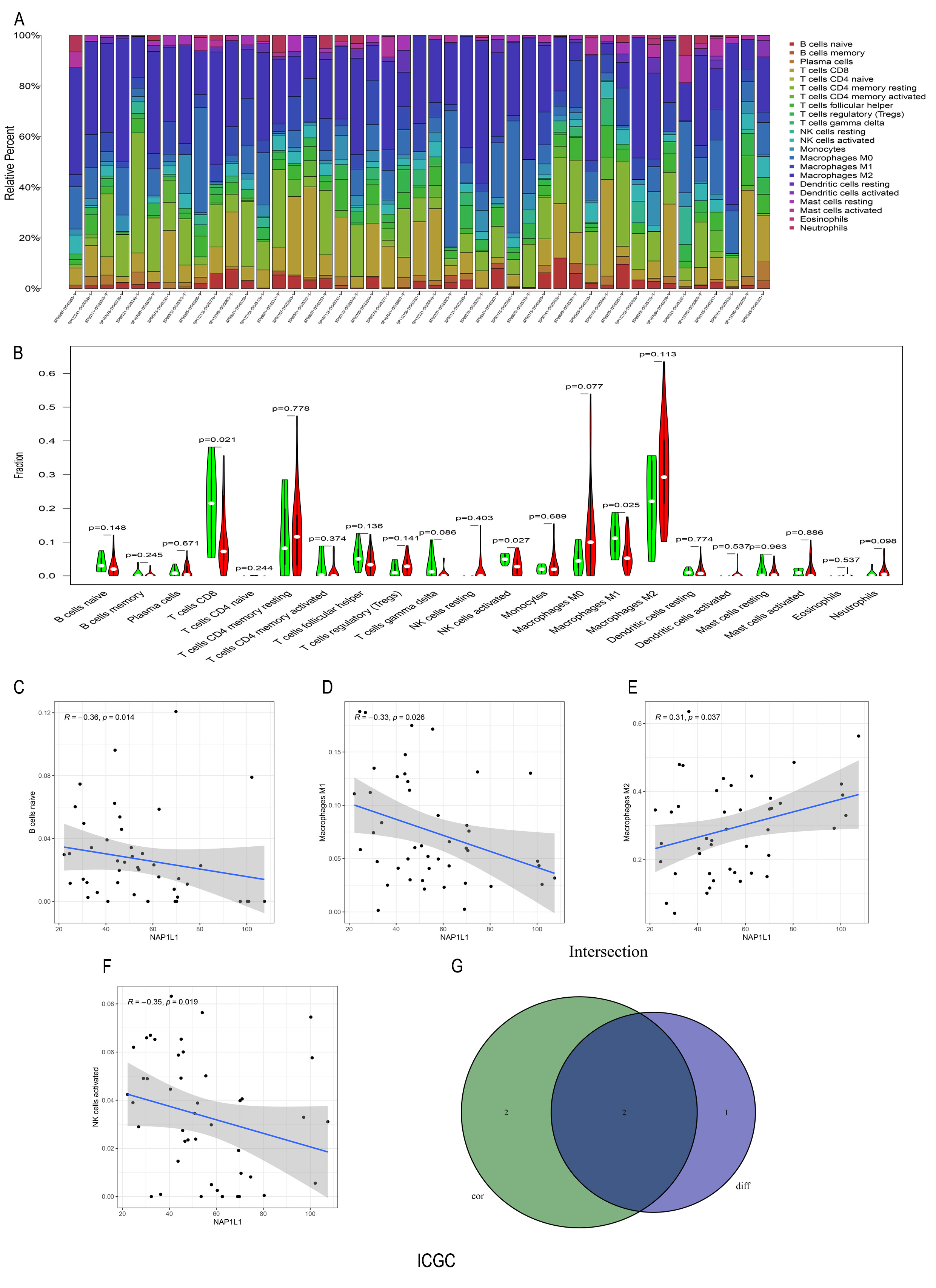

Supplement: Supplementary file 2 [file DataSheet2.ZIP › supplementary figure/Supplement Figure 6.jpg]

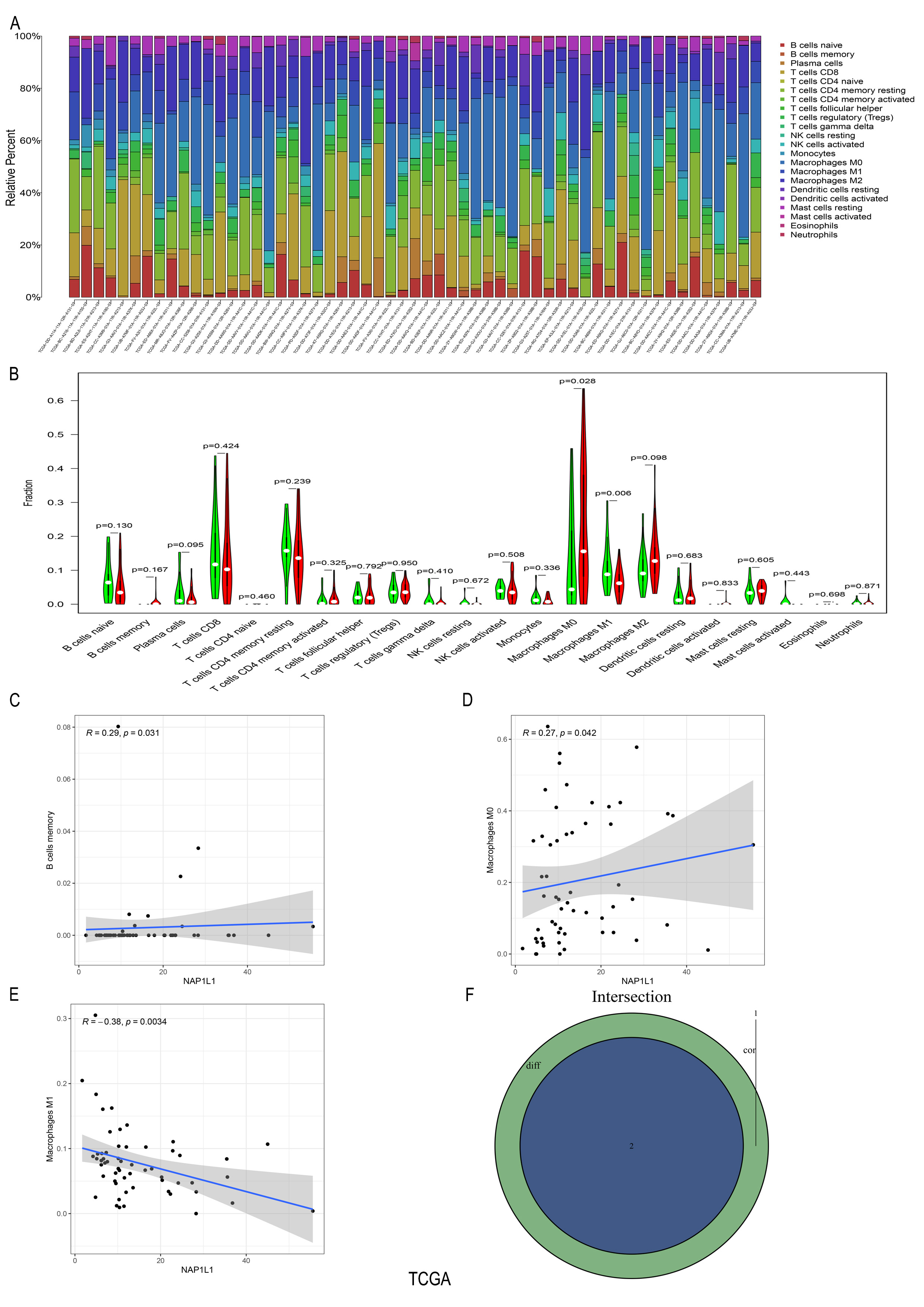

Supplement: Supplementary file 2 [file DataSheet2.ZIP › supplementary figure/Supplement Figure 7.jpg]
