# Supplementary material for: NAP1L1 Functions as a Novel Prognostic Biomarker Associated With Macrophages and Promotes Tumor Progression by Influencing the Wnt/β-Catenin Pathway in Hepatocellular Carcinoma
Source: Front Genet. 2022 May 19;13:876253. doi: 10.3389/fgene.2022.876253 (PMC9161088; doi:10.3389/fgene.2022.876253)
Supplement: Supplementary file 2 [file DataSheet2.ZIP › supplementary figure/Supplement Figure legends.docx]

**Supplement Figure 1**

The survival analysis, the clinical features analysis, the univariate/multivariate regression analysis, and the multiple GSEA analysis in the GSE76427 datasets

**(A)** The scatter plot of the *NAP1L1* expression in the non-tumor and HCC specimens.

**(B)** The survival curve of DFS in the high and the low *NAP1L1* expression groups.

**(C)** The bar graph in age

**(D)** The bar graph **in gender.**

**(E)** The **bar graph in stage.**

**(F)** The **univariate regression analysis.**

**(G)** The **multivariate regression analysis.**

**(H)** The multiple **GSEA analysis** ground on **the GO.**

**(I)** The **multiple GSE**A analysis ground on **the** KEGG.

**Supplement Figure 2**

The survival analysis, the clinical features analysis, the univariate/multivariate regression analysis, and the multiple GSEA analysis in the ICGC datasets

**(A)** The scatter plot of the *NAP1L1* expression in the non-tumor and HCC specimens.

**(B)** The survival curve of OS in the high and the low *NAP1L1* expression groups.

**(C)** The bar graph in age

**(D)** The bar graph **in gender.**

**(E)** The **bar graph in stage.**

**(F)** The **univariate regression analysis.**

**(G)** The **multivariate regression analysis.**

**(H)** The multiple **GSEA analysis** ground on **the GO.**

**(I)** The **multiple GSE**A analysis ground on **the** KEGG.

**Supplement Figure 3**

The survival analysis, the clinical features analysis, the univariate/multivariate regression analysis, and the multiple GSEA analysis in TCGA datasets

**(A)** The scatter plot of the *NAP1L1* expression in the non-tumor and HCC specimens.

**(B)** The survival curve of OS in the high and the low *NAP1L1* expression groups.

**(C)** The bar graph in age

**(D)** The bar graph **in gender.**

**(E)** The **bar graph in stage.**

**(F)** The **univariate regression analysis.**

**(G)**The **multivariate regression analysis.**

**(H)** The multiple **GSEA analysis** ground on **the GO.**

**(I)** The **multiple GSE**A analysis ground on **the** KEGG.

**Supplement Figure 4**

**The** Western blot and **the** flow cytometry

**(A) The** Western blot was utilized to examine the expression of *NAP1L1*, *CDK1*, and *GAPDH*.

**(B) The** Flow cytometric was utilized to count **the cell number of the cell cycle** in MHCC-97H and Huh7 cells.

**(C) The** si-1 and si-2 groups promoted **the** cell cycle arrest at the G2 phase ground on **the** NC group. (**p* < 0.05; ***p* < 0.01; ****p* < 0.001).

**Supplement Figure 5**

**The** analysis of immune infiltration in the GSE76427 dataset.

**(A)** **The** bar graph containing **the** 22 immune cells.

**(B) The** violin graph containing the 22 immune cells.

**(C)** The relationship of *NAP1L1* and M0 Macrophages.

**(D)** The relationship of *NAP1L1* and gamma delta T cells.

**(E)** The Venn graph was utilized to picture the meaningful immune cells between the violin plot and the correlation graph.

**Supplement Figure 6**

**The** analysis of immune infiltration in ICGC dataset.

**(A)** **The** bar graph containing **the** 22 immune cells.

**(B) The** violin graph containing the 22 immune cells.

**(C)** The relationship of *NAP1L1* and naive B cells.

**(D)** The relationship of *NAP1L1* and M1 Macrophages.

**(E)** The relationship of *NAP1L1* and M2 Macrophages.

**(F)** The relationship of *NAP1L1* and activated NK cells.

**(G)** The Venn graph was utilized to picture the meaningful immune cells between the violin plot and the correlation graph.

**Supplement Figure 7**

**The** analysis of immune infiltration in TCGA dataset.

**(A) The** bar graph containing **the** 22 immune cells in each sample.

**(B) The** violin graph containing the 22 immune cells.

**(C)** The relationship of *NAP1L1* and memory B cells.

**(D)** The relationship of *NAP1L1* and M0 Macrophages.

**(E)** The relationship of *NAP1L1* and M1 Macrophages.

**(F)** The Venn graph was utilized to picture the meaningful immune cells between the violin plot and the correlation graph.
